# Functional Dissection of the *Drosophila melanogaster* Condensin Subunit Cap-G Reveals Its Exclusive Association with Condensin I

**DOI:** 10.1371/journal.pgen.1003463

**Published:** 2013-04-18

**Authors:** Sabine Herzog, Sonal Nagarkar Jaiswal, Evelin Urban, Anna Riemer, Sina Fischer, Stefan K. Heidmann

**Affiliations:** 1 Lehrstuhl für Genetik, University of Bayreuth, Bayreuth, Germany; 2 Lehrstuhl für Pflanzenphysiologie, University of Bayreuth, Bayreuth, Germany; Stowers Institute for Medical Research, United States of America

## Abstract

The heteropentameric condensin complexes have been shown to participate in mitotic chromosome condensation and to be required for unperturbed chromatid segregation in nuclear divisions. Vertebrates have two condensin complexes, condensin I and condensin II, which contain the same structural maintenance of chromosomes (SMC) subunits SMC2 and SMC4, but differ in their composition of non–SMC subunits. While a clear biochemical and functional distinction between condensin I and condensin II has been established in vertebrates, the situation in *Drosophila melanogaster* is less defined. Since *Drosophila* lacks a clear homolog for the condensin II–specific subunit Cap-G2, the condensin I subunit Cap-G has been hypothesized to be part of both complexes. *In vivo* microscopy revealed that a functional Cap-G-EGFP variant shows a distinct nuclear enrichment during interphase, which is reminiscent of condensin II localization in vertebrates and contrasts with the cytoplasmic enrichment observed for the other EGFP-fused condensin I subunits. However, we show that this nuclear localization is dispensable for Cap-G chromatin association, for its assembly into the condensin I complex and, importantly, for development into a viable and fertile adult animal. Immunoprecipitation analyses and complex formation studies provide evidence that Cap-G does not associate with condensin II–specific subunits, while it can be readily detected in complexes with condensin I–specific proteins *in vitro* and *in vivo*. Mass-spectrometric analyses of proteins associated with the condensin II–specific subunit Cap-H2 not only fail to identify Cap-G but also the other known condensin II–specific homolog Cap-D3. As condensin II–specific subunits are also not found associated with SMC2, our results question the existence of a soluble condensin II complex in *Drosophila*.

## Author Summary

The accurate duplication and segregation of chromosomes during cell divisions are prerequisites for ensuring genetic stability within an individual organism and in entire populations. Among the many components involved in regulating these processes, a protein complex called condensin plays a crucial role in shaping mitotic chromosomes, so that they can be faithfully distributed. Many organisms contain two of these condensin complexes (condensin I and II), which both have been shown to be required for accurate chromosome distribution. In the fly *Drosophila melanogaster*, condensin II appears to lack one of its components, called Cap-G2. We have tested the hypothesis whether the corresponding component of condensin I (Cap-G) might also participate in the assembly of condensin II. Careful analyses of complexes formed in the living organism or in the test tube argue against Cap-G being part of condensin II. Moreover, our results question the very existence of a soluble condensin II complex in flies, as opposed to other organisms. Surprisingly, a substantially truncated variant of the essential Cap-G still supports development of living and fertile flies. As this variant localizes within the cell differently from full-length Cap-G, our results show that localization of a protein does not always determine its function.

## Introduction

Chromosome condensation is a critical process ensuring faithful distribution of the replicated genetic information onto the daughter cells. While the exact mechanism underlying the longitudinal compaction of the dispersed interphase chromatin into the rod-like and sturdy metaphase chromosomes is still subject of intense research, the participation of the condensin complexes in this process has been thoroughly demonstrated (for review see [1]–[3]). However, while condensin is clearly required and sufficient for compaction of sperm chromatin incubated in *Xenopus laevis* egg extracts [4], [5], the phenotypes observed after condensin depletion in other systems suggest the existence of alternative mechanisms mediating chromatin compaction. Condensin depletion in vertebrate cells, worms and flies does affect the structure of mitotic chromosomes, but compaction of chromatin is only slightly impaired. The extent of this compaction phenotype varies by the organism studied and the experimental system used (for review see [3]). However, in all cases, persistent interconnections of chromatin fibres can be observed in anaphase (so-called anaphase bridges), resulting in severe problems during chromatid segregation in mitosis. Thus, condensin has a role in resolving chromatin bridges present between the replicated chromatids.

Plants and animals harbour two condensin complexes, both containing the structural maintenance of chromosomes (SMC) proteins SMC2 and SMC4, but differing in their non-SMC regulatory subunits. Condensin I complexes contain the subunits Cap-D2, Cap-G and Cap-H (also called Barren in *Drosophila*), while condensin II complexes contain the related subunits Cap-D3, Cap-G2 and Cap-H2. Cap-H and Cap-H2 belong to the kleisin family of proteins which are characterized by their ability to bind to the head domains of SMC protein dimers [6]. Cap-G, Cap-G2, Cap-D2 and Cap-D3 contain in their N-terminal parts extended regions of Huntingtin, elongation factor 3, A-subunit of protein phosphatase 2A, TOR1 lipid kinase (HEAT) repeats, which are thought to mediate protein-protein interactions [7]. In vertebrates, both condensin complexes play essential roles and collaborate in structuring of mitotic chromosomes and in ensuring their unperturbed segregation. Interestingly, the two complexes fulfil non-overlapping functions as exemplified by distinct phenotypes upon depletion of either condensin I or condensin II-specific subunits [8]–[10], by their alternating association with mitotic chromosomes [11], [12], or by their different localization in interphase cells: Condensin I-specific subunits are enriched in the cytoplasm, while condensin II-specific subunits can be found primarily in the nucleus [9]–[11]. Within the eukaryotic kingdom, the composition of the condensin complexes found in different species is not uniform. Fission and budding yeast harbour homologs only for condensin I, as do e.g. ciliates and kinetoplastids (for review see [3]). *C. elegans*, on the other hand, contains three condensin complexes, one of which (condensin I^DC^) has specialized to function in dosage compensation in hermaphrodites [13]. In *Drosophila melanogaster*, condensin I is present, but for condensin II only the subunits Cap-H2 and Cap-D3 can be identified. No gene encoding the condensin II-specific subunit Cap-G2 is apparent in the genome. This has led to the speculation that *Drosophila* Cap-G might be a component of both complexes, just as SMC2 and SMC4 [14]–[16]. The essential role for all condensin I-specific subunits in mitotic proliferation is well established [14], [17]–[22]. On the other hand, loss-of-function mutations of the *Drosophila* genes encoding Cap-H2 and Cap-D3 are viable, indicating that their function is dispensable for mitotic proliferation [18], [23], [24]. However, *Cap-D3* and *Cap-H2* mutant males are sterile, and cytological as well as genetic evidence clearly indicates a role during male meiosis for these two subunits [23]. Interestingly, mutations in *Cap-H2* have also been shown to prevent the dispersal of nurse cell polytene chromosomes, which are present for a short developmental period during oogenesis, and to enhance transvection phenomena. Conversely, *Cap-H2* overexpression leads to dispersal of the polytene chromosomes in larval salivary glands and in addition suppresses transvection [24]. These results suggest that Cap-H2 negatively regulates chromosome associations and additional genetic evidence indicates that this function is dependent on Cap-D3 [24]. Moreover, Cap-D3 has been shown to interact with the *Drosophila* Retinoblastoma (Rb)-protein homolog Rbf and the two proteins colocalize on the regulatory regions for transcription of the antimicrobial peptide (AMP) genes, thereby influencing innate immunity [16], [25]. Thus, the *Drosophila* condensin II subunits Cap-H2 and Cap-D3 perform roles in regulating gene expression, as has been demonstrated for condensin complexes in other studies [20], [21], [26], [27]. However, whether these functions are performed in the context of a physical protein complex containing SMC2, SMC4, Cap-H2, Cap-D3 and possibly Cap-G is unknown. While biochemical evidence for the existence of a soluble condensin I complex has been published [18], the existence and protein composition of a soluble condensin II-like complex in *Drosophila* is uncertain.

Here, we have analyzed in detail the localization behaviour and complex formation capabilities of *Drosophila* Cap-G *in vivo* and *in vitro* to test the hypothesis, whether it might be a common component of both condensin complexes in *Drosophila*. The comparison of the localization and dynamics of various fluorescently tagged, functional condensin subunits highlights the fact that Cap-G indeed behaves differently from other condensin I-complex components. However, complex formation studies strongly argue against Cap-G being associated with condensin II-specific components. Furthermore, immunoprecipitation analyses consistently provide evidence for soluble condensin I complexes, but fail to support the presence of native soluble condensin II complexes *in vivo* and indicate a strongly reduced complex formation potential *in vitro*. Thus, while we cannot exclude the assembly of condensin II-like complexes specifically on chromatin in specialized cell types, our data argue against the existence of an abundant and stable soluble condensin II complex in *Drosophila*.

## Results

### Localization of *Drosophila* condensin subunits during the cell cycle

In interphase, vertebrate condensin I subunits are primarily cytoplasmic, while condensin II subunits are primarily nuclear [9]–[11]. Consistently, *Drosophila* Barren/Cap-H and Cap-H2 have also been found to be cytoplasmic or nuclear enriched, respectively [24], [28]. Towards a comparative description of the localization behavior of *Drosophila* condensin subunits in the living organism, we have generated EGFP-fused variants of the condensin subunits Cap-D2, SMC2 and Cap-G (Figure S1A). EGFP-Cap-D2 should label exclusively condensin I-complexes, while SMC2_h_-EGFP is expected to occur in both condensin I and condensin II. As no condensin II-specific Cap-G2 subunit has been identified in *Drosophila*, Cap-G has been hypothesized to be also part of both condensin complexes [14]–[16]. Thus, Cap-G localization may provide a hint as to whether it is part of only condensin I or both condensin complexes in *Drosophila*.

**Figure 1:**
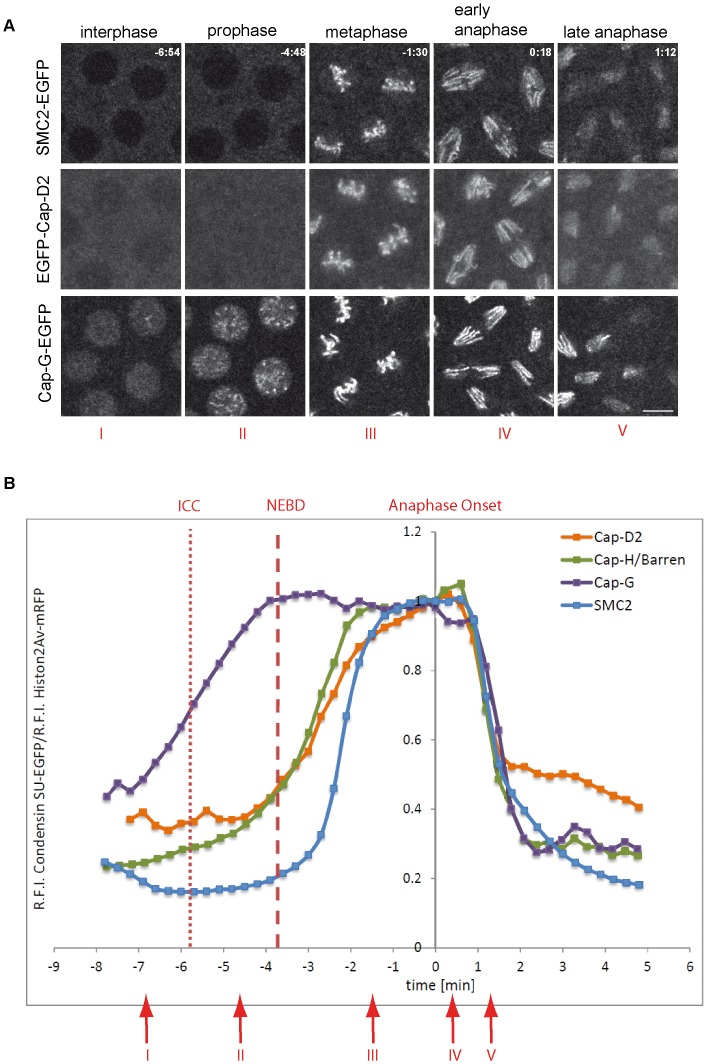
Localization of *Drosophila* condensin subunits during the cell cycle. (A) Living embryos expressing *gHis2Av-mRFP1* together with *gSMC2_h_-EGFP* (upper panel), *gEGFP-Cap-D2* (middle panel), or *gCap-G-EGFP* (lower panel) were imaged while progressing through nuclear cycle 12. Representative images for indicated time points in min are shown (t = 0.0 min, anaphase onset). Note that SMC2_h_-EGFP and EGFP-Cap-D2 are enriched in the cytoplasm during interphase, while Cap-G-EGFP is nuclear enriched. Scale bar is 5 µm. (B) Fluorescence intensities of EGFP-Cap-D2 (orange), Cap-G-EGFP (purple), and SMC2_h_-EGFP (blue) were determined for selected nuclei in each frame and are plotted as relative intensities per nucleus. Data series were aligned accordingly to anaphase onset (t_0_ = last metaphase). Data sets from a total of 15 to 37 nuclei from seven to 15 embryos were aligned. The values for Cap-H/Barren (green) are taken from Oliveira et al., 2007 [28]. The times of initiation of chromatin condensation (ICC) and NEBD are indicated by the dotted and dashed red lines, respectively. The red arrows with the roman type numerals correspond to the images shown in (A).

All three transgene constructs are expressed under control of the flanking genomic regulatory sequences and quantification of the expression levels in early embryogenesis reveal a ratio of transgene products of approximately 1∶4∶8 (Cap-G-EGFP∶SMC2_h_-EGFP∶EGFP-Cap-D2; Figure S1B). Despite these differences, all transgenes encode biologically functional products as the presence of single copies of the transgenes can complement the lethality associated with loss-of-function-mutations in the respective genes (Table S1 and data not shown). Analysis of living embryos progressing through the divisions of the syncytial blastoderm revealed that during interphase, SMC2_h_-EGFP and EGFP-Cap-D2 are enriched in the cytoplasm, as has been reported previously for the condensin I - specific subunit Cap-H/Barren (Figure 1A; Videos S1 and S2; [28]). In contrast, Cap-G-EGFP is nuclear enriched in interphase, reminiscent of condensin II localization in vertebrates (Figure 1A, Video S3). All three EGFP-fused subunits rapidly associate with condensing chromatin at early stages of mitosis. However, Cap-G-EGFP associates with chromatin slightly earlier than EGFP-Cap-D2 and SMC2_h_-EGFP, which might be due to its preferential nuclear localization in interphase. All condensin subunits leave chromatin during late anaphase/early telophase (Figure 1A; Videos S1, S2, S3). As the different condensin subunits exhibit distinct localization patterns during interphase, and differ in their chromatin association kinetics, we scrutinized the dynamics of mitotic chromatin association of these subunits during cycle 12 of the syncytial divisions. To this end, we performed quantitative measurements of the EGFP fluorescence signals and normalized them to the simultaneously recorded fluorescence measurements of the mRFP1-fused histone variant His2Av, which was also expressed in these embryos. The data revealed that Cap-G-EGFP is loaded maximally already at nuclear envelope breakdown, a time-point when the EGFP-fused subunits Cap-D2 and Cap-H/Barren (data from [28]) are just beginning to associate with chromatin (Figure 1B). Interestingly, SMC2_h_-EGFP loading appears even more delayed (half-maximal association of SMC2 is −2.5 min before anaphase onset vs. −3.5 min for Cap-D2 and Cap-H/Barren; Figure 1B). Similar loading kinetics are observed, when SMC2_h_-EGFP chromatin association was determined in an *SMC2* mutant background, ruling out the possibility that the presence of endogenous SMC2 significantly delays incorporation of the EGFP-fused variant (Figure S2). For all analyzed subunits, maximal chromatin association levels are achieved during late metaphase/early anaphase. During exit from mitosis, the four condensin subunits delocalize from chromatin with almost identical kinetics (Figure 1B).

**Figure 2:**
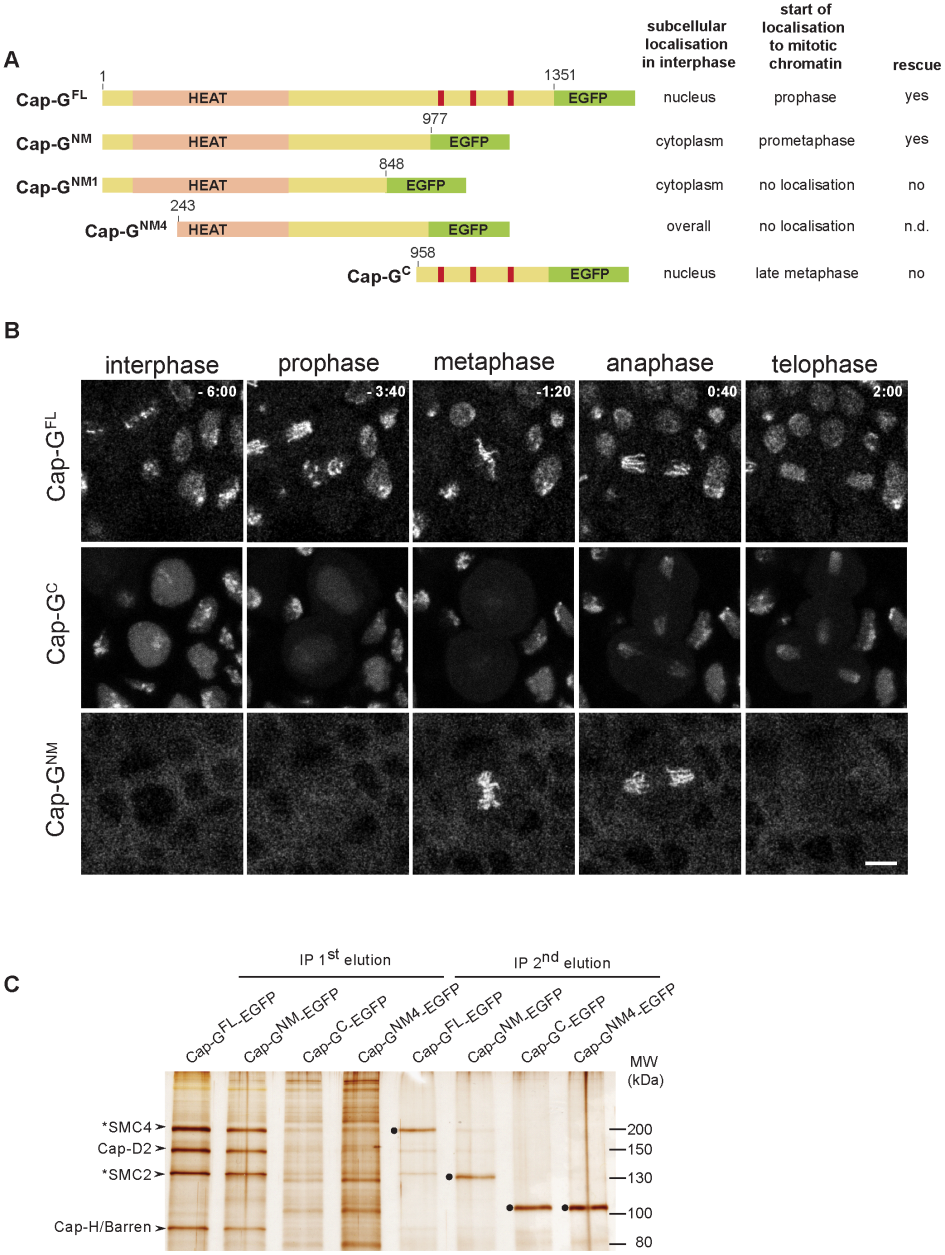
The N-terminal two-thirds of Cap-G are sufficient for interaction with chromatin and condensin I subunits. (A) Left panel. Schematic drawing of the analyzed EGFP-fused Cap-G-fragments. Full-length Cap-G (Cap-G^FL^) encompasses 1351 aa. HEAT-repeats (pale pink) are predicted by SMART between residues 50 and 553, and nuclear import signals (red bars) are predicted by PSORT at aa positions 1072, 1162 and 1210. Right panel. The localization characteristics of the Cap-G constructs as well as their ability to complement the lethality associated with *Cap-G* loss-of-function mutants is indicated. (B) Subcellular localization and chromatin association of different EGFP-fused Cap-G-fragments observed in living embryos progressing through epidermal mitosis14. Expression of different *UAST-Cap-G-EGFP* transgenes was driven by *α4-tub-GAL4-VP16*. Cap-G^C^-EGFP is enriched inside the nuclei during interphase but does not associate with chromatin during early stages of mitosis. In contrast, Cap-G^NM^-EGFP is mainly cytoplasmic during interphase and associates with mitotic chromatin immediately after NEBD. Individual frames of representative time lapse movies are shown with time points indicated in min (t = 0, anaphase onset). Scale bar is 5 µm. (C) Extracts from 3–6 h old embryos expressing various EGFP-fused Cap-G-fragments driven by *α4-tub-GAL4-VP16* were subjected to immunoprecipitation with rabbit-anti-EGFP antibodies. Bound proteins were eluted in two steps with increasing stringency. Precipitates were separated by SDS-PAGE and subjected to silver staining. The identity of Cap-H/Barren and Cap-D2 was confirmed by immunoblotting, SMC2 and SMC4 were assigned according to their expected molecular weight (indicated by asterisks). The condensin I subunits were efficiently precipitated by both Cap-G^FL^-EGFP and Cap-G^NM^-EGFP and were eluted during the first step (IP 1^st^ elution), while they were not significantly precipitated by Cap-G^NM4^-EGFP and Cap-G^C^-EGFP. The second elution step (IP 2^nd^ elution) mainly reveals the recovery of the EGFP-fused Cap-G-fragments (filled circles). Note that Cap-G^FL^-EGFP and Cap-G^NM^-EGFP migrate at the same position in the SDS-polyacrylamide gel as SMC4 and SMC2, respectively.

To assess, which regions of Cap-G mediate the subcellular localization during the cell cycle, we expressed various EGFP-fused deletion constructs under GAL4/UAS-control in early embryos and analyzed the localization behavior of the fusion proteins while cells were progressing through epidermal mitosis 14 (Figure 2A). Computational analyses predict nuclear localization signals (NLS) at positions 1072, 1162, and 1210. Consistently, a C-terminal Cap-G fragment (Cap-G^C^; aa 958–1351) encompassing these signals is strongly nuclear enriched in interphase. At nuclear envelope breakdown, the fusion protein distributes throughout the cell (Figure 2B, Video S4). During early to mid mitosis, Cap-G^C^-EGFP associates only very weakly with chromatin. However, beginning with late anaphase, Cap-G^C^-EGFP accumulates on the segregating chromatids (Figure 2B, Video S4). The construct Cap-G^NM^-EGFP (aa 1–977) lacks the C-terminal region with the NLS, but retains an extended region predicted to form HEAT-repeats and it displays a complementary localization behavior when compared to Cap-G^C^-EGFP. In interphase, this Cap-G variant is primarily localized in the cytoplasm, but approximately 20–40 sec after nuclear envelope breakdown, it associates rapidly and efficiently with mitotic chromatin (Figure S3). Starting with anaphase, Cap-G^NM^-EGFP dissociates from chromatin similar to full length Cap-G^FL^-EGFP (Figure 2B, Video S5) and as was observed for the other condensin subunits (Figure 1B; [28]).

To assess, whether the mitotic localization behavior of Cap-G^NM^-EGFP reflects its potential to form complexes with the other condensin subunits, we performed immunoprecipitation analyses. Extracts were prepared from embryos expressing various EGFP-fused Cap-G variants followed by precipitation using anti-EGFP antibodies. Proteins bound to the beads were eluted in two steps, with the second being more stringent. Four prominent protein bands in the high molecular weight range can be detected on silver stained gels in the first round eluates of both Cap-G^FL^-EGFP and Cap-G^NM^-EGFP-coupled beads (Figure 2C). The identity of two of the bands was confirmed as Cap-D2 and Cap-H/Barren by immunoblot analysis (Figure S4). Based on their migration behavior, the first and third bands were suspected to correspond to SMC4 and SMC2, respectively. This assignment was corroborated by mass-spectrometric analyses of Cap-G^FL^-EGFP immunoprecipitates (see below). The antibody-bound EGFP-fused Cap-G variants were primarily eluted under more stringent conditions (Figure 2C). Cap-G^C^-EGFP immunoprecipitates did not contain the other condensin I subunits in significant amounts, as did not the precipitates of a Cap-G^NM^-EGFP- variant with a further N-terminal truncation of 242 amino acids (Cap-G^NM4^-EGFP). This latter variant does not localize to mitotic chromatin and it is distributed in interphase throughout the cell (Figure S5). The HEAT repeats predicted to form in the N-terminal region of Cap-G are implicated in protein-protein interactions [7]. Thus, binding of Cap-G to the condensin complex may be mediated via the HEAT-repeat motifs, since Cap-G^NM4^-EGFP lacking a large part of this domain is not able to precipitate Cap-D2 or Cap-H/Barren. However, the N-terminal 242 amino acids are not sufficient for efficient association with mitotic chromatin, since the variant Cap-G^NM1^-EGFP, which encompasses the region of aa 1–848, is primarily cytoplasmic in interphase like Cap-G^NM^-EGFP, and associates only very weakly with chromatin during mitosis (Figure S5). We conclude that the C-terminal third of Cap-G contains nuclear localization sequences, but it is dispensable for mitotic chromatin association. Moreover, the HEAT-repeat region as well as the stretch encompassing aa 848–977 within the N-terminal two-thirds of Cap-G are required for binding to mitotic chromatin, most probably by virtue of their mediating the assembly into condensin complexes.

### Cap-G-EGFP co-localizes in interphase with HP-1 and initiates chromatin loading at centromeres

**Figure 3:**
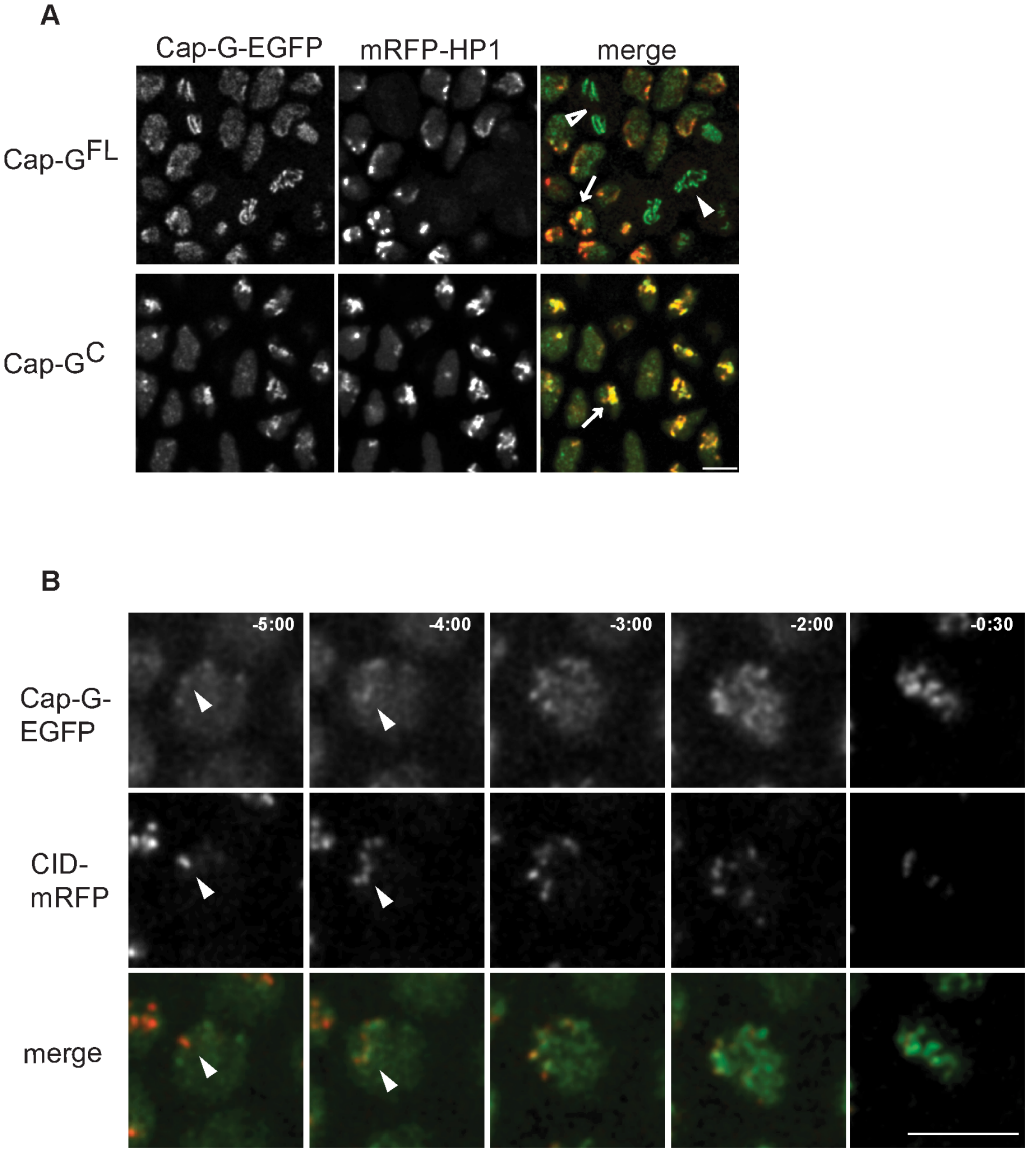
Cap-G-EGFP co-localizes with HP1 in interphase and initiates chromatin loading at centromeres. (A) Cap-G is enriched at heterochromatic regions during interphase. Living embryos co-expressing *UAST-Cap-G^FL^-EGFP* or *UAST-Cap-G^C^-EGFP* (green in merged panels) and *mRFP1-HP1* (red in merged panels) were analyzed while progressing through epidermal mitosis 14. Both EGFP-fused Cap-G-variants are locally enriched within interphase nuclei and show a particular co-localization with mRFP1-HP1 (arrows). Cap-G^FL^-EGFP localizes to the chromatin in metaphase (filled arrowhead) and anaphase cells (open arrowhead). (B) Cap-G loading initiates at centromeres. Embryos co-expressing *gCap-G^FL^-EGFP* (green in merged panels) and *Cid-mRFP* (red in merged panels) were analyzed to determine the initial sites of Cap-G^FL^-EGFP loading while progressing through post-blastodermal mitosis 14. Individual frames of a representative single nucleus are shown, with indicated times in min∶sec (t = 0, anaphase onset). Early Cap-G^FL^-EGFP accumulations frequently co-localize with Cid-mRFP1 signals (arrowheads). Scale bar 5 µm.

We have noticed that during interphase, Cap-G^FL^-EGFP and Cap-G^C^-EGFP are not homogeneously distributed in the nucleoplasm. As the patchy appearance of Cap-G signals is reminiscent of heterochromatin distribution in these nuclei, we analyzed embryos expressing EGFP-fused Cap-G^FL^ or Cap-G^C^ concomitant with a red fluorescently labeled variant of heterochromatin protein 1 (mRFP1-HP1) (Figure 3A). HP1 binds to histone H3 methylated at lysine 9 and is thus a marker for heterochromatin distribution in interphase cells [29]. *In vivo* microscopy of embryos progressing through epidermal cycle 14 revealed that the two Cap-G variants indeed largely co-localize with mRFP1-HP1 during interphase, indicating heterochromatin association of Cap-G (Figure 3A). During mitosis, mRFP1-HP1 dissociates from chromatin, as has been previously observed with fixed material (arrowheads in Figure 3A; [30]). This observation, together with the fact that Cap-G^C^ associates with chromatin in late mitosis when mRFP1-HP1 is still absent, indicates that Cap-G chromatin association does not depend on the presence of HP1. While Cap-G clearly co-localizes with heterochromatin in interphase, it does not appear to be physically associated with HP1 in a common protein complex as HP1 cannot be co-precipitated with Cap-G (Figure S6).

Embedded within the heterochromatin are the centromeres. As enrichment of other condensin subunits in centromeric regions has been demonstrated [11], [17], [31]–[33] and a genetic and physical interaction of Cap-G with the centromeric H3 variant Cid has been established [14], we scrutinized the dynamics of Cap-G chromatin association. To this end, we analyzed the localization behavior of Cap-G^FL^-EGFP in comparison with Cid-mRFP1 in embryos progressing through cycle 14. Indeed, early chromatin accumulation of Cap-G^FL^-EGFP occurs in nuclear regions where Cid-mRFP1 signals can be detected (Figure 3B). Similar dynamics are observed when embryos progress through syncytial cycle 12, and quantitation reveals an approximately twofold enrichment of Cap-G^FL^-EGFP in centromere-proximal vs. centromere-distal regions in early stages of Cap-G chromatin association (Figure S7). Thus, our observations are consistent with a model in which Cap-G first binds to centromeric regions and then spreads into the adjacent heterochromatin.

### Interphase nuclear localization of Cap-G is dispensable for condensin function during the cell cycle and development

The C-terminus of Cap-G is required for nuclear localization and sufficient to confer heterochromatic enrichment during interphase. The N-terminal two-thirds of Cap-G, on the other hand, are sufficient for efficient chromatin localization during mitosis and for assembly within the condensin I holocomplex. To assess the relevance of the functional features contributed by the Cap-G C-terminus, we generated individuals expressing Cap-G^NM^ or Cap-G^NM^-EGFP as sole source for this condensin subunit in a *Cap-G^1^/Cap-G^6^ trans*-heterozygous mutant background. Loss-of-function mutations in *Cap-G* are embryonic lethal [14], [20]. Expression of *Cap-G^FL^-EGFP*, either under control of the genomic regulatory sequences or under GAL4/UAS control using the ubiquitous *da-GAL4* driver, gave rise to viable and fertile adults demonstrating the biological functionality of these constructs (Table S1). Surprisingly, adult flies were also obtained with high efficiency by ubiquitous expression of two independent pUAST-based *UAS-Cap-G^NM^-EGFP*-transgene insertions in the same *trans*-heterozygous *Cap-G* mutant background. As pUAST does not direct expression in the female germline, female fertility could not be assessed in these cases. However, expression from the *Cap-G^NM^-EGFP* transgene contained in a pUASP-based vector, which also allows expression in the female germline [34], restored fertility in both sexes (Table S1). Immunoblot analysis confirmed that these animals lacked expression of endogenous *Cap-G* and survived solely due to the expression of the C-terminally truncated Cap-G variants (Figure S8A). To assess, whether the C-terminally truncated Cap-G^NM^ variant also fails to localize to interphase nuclei in the absence of competing full-length Cap-G, we analyzed Cap-G^NM^-EGFP localization in the rescue situation. Cap-G^NM^-EGFP is excluded from the nuclei in interphase also in a *Cap-G* mutant background, and it does not bind to chromatin in prophase, ruling out the possibility that the presence of competing full-length Cap-G might prevent early chromatin association of the Cap-G^NM^ variant (Figure S8B and see also Figure S10C). Not all Cap-G^NM^ transgenes complemented the *Cap-G* mutant phenotype efficiently. Fertility was only observed after crosses of rescued individuals with wild type flies, and many eggs laid by Cap-G^NM^ rescued mothers displayed developmental defects (data not shown). Therefore, it was not possible to establish stable rescue stocks. We conclude nevertheless that the C-terminal 374 amino acids of Cap-G are not absolutely critical for condensin function required for development from the fertilized egg to a fertile adult. While the full-length protein rescues with higher efficiency than the C-terminal truncated version when expressed at comparable levels (Table S1; genomic transgenes), the development of fertile adult animals is still possible when the C-terminal domain of Cap-G is lacking. As this C-terminal part contains the NLS, nuclear enrichment of Cap-G during interphase is dispensable for condensin function in the cell cycle and during development.

### Cap-G is not associated with condensin II–specific subunits *in vivo*

**Figure 4:**
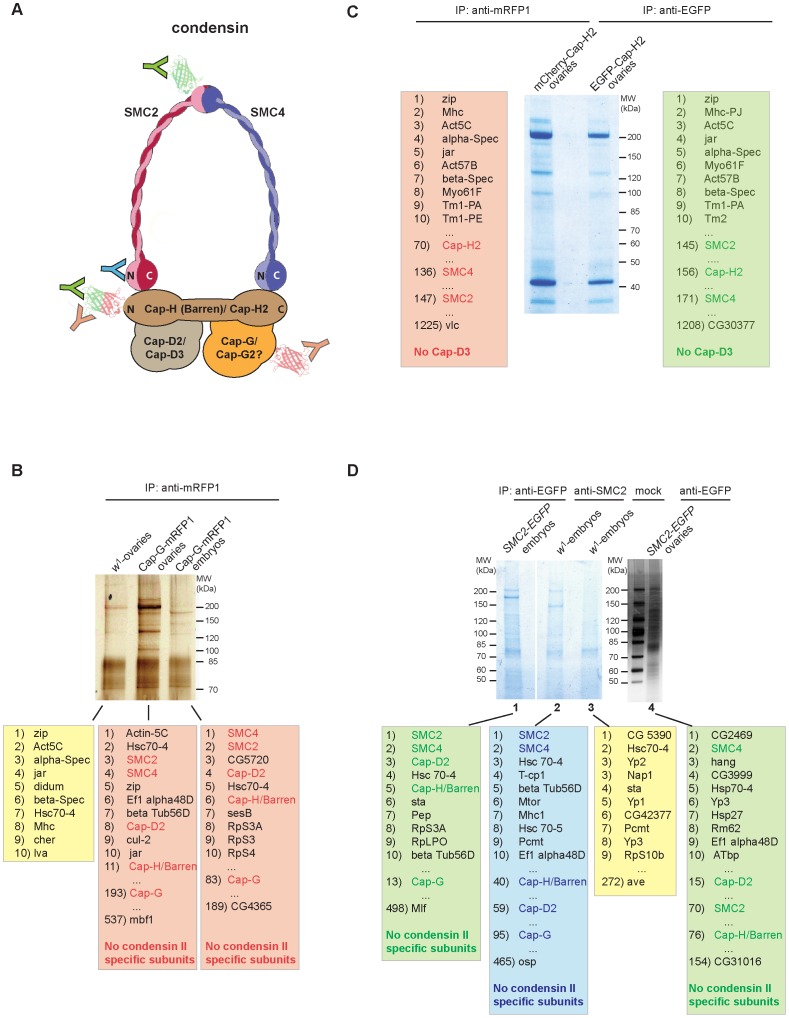
Cap-G is not associated with condensin II–specific subunits *in vivo*. (A) Schematic model of a condensin complex with the positions indicated that are recognized by antibodies used in the immunoprecipitation experiments. Fused EGFP or mCherry/mRFP tags are depicted as green or red barrel-like structures, respectively. The red/green colored barrel fused to the N-terminus of Cap-H2 indicates that fusions with both EGFP and mCherry were analyzed as shown in panel (C). Color coding of the antibodies (Y) corresponds to the color shading of the boxes with the lists of identified proteins within the precipitates shown in panels (B–D). (B) Protein extracts from 3–6 h old embryos expressing *gmRFP1-Cap-G* as well as from ovaries of wild type females (*w^1^*) or females expressing *gmRFP1-Cap-G* were subjected to immunoprecipitation with anti-mRFP antibodies. Precipitated proteins were separated by SDS-PAGE and visualized by silver staining and in a parallel experiment with colloidal Coomassie Blue. Coomassie Blue stained lanes were processed for mass spectrometric (MS) analysis. (C) Ovary extracts derived from females expressing *UASP1-mCherry-Cap-H2* or *UASP1-EGFP-Cap-H2* driven by *tubP-Gal4* were subjected to immunoprecipitation with anti-mRFP1 antibodies. Immunoprecipitates were separated by SDS-PAGE, stained with colloidal Coomassie Blue and processed for MS analysis. (D) Protein extracts from 3–6 h old embryos expressing *gSMC2_h_-EGFP* (lane 1) or wild type embryos (lanes 2 and 3) as well as from ovaries of females expressing *gSMC2_h_-EGFP* (lane 4) were subjected to immunoprecipitation using anti-EGFP antibodies (lanes 1 and 4), anti-SMC2-antibodies (lane2) or were mock treated with beads only (lane 3). Immunoprecipitates were separated by SDS-PAGE, stained with colloidal Coomassie Blue (lanes 1–3) or a silver stain (lane 4) and processed for MS analysis directly (lanes 1–3) or after a parallel SDS-PAGE subsequently stained with colloidal Coomassie (lane 4). In each case, the list of identified *Drosophila* proteins was sorted according to the cumulative peptide intensities. The top ten ranked proteins, condensin subunits, and the lowest ranked entries are listed.

Due to the lack of an obvious Cap-G2 homolog encoded in the *Drosophila* genome, Cap-G has been hypothesized to be part of both condensin subunits, just as SMC2 and SMC4 [14]–[16]. In the anti-Cap-G^FL^-EGFP immunoprecipitates shown in Figure 2C, four prominent high molecular weight bands are evident, which were assigned to the condensin I-specific subunits and the two SMC's. As the condensin II-specific subunits Cap-H2 and Cap-D3 might not have been abundant enough in the analyzed extracts to be detected by silver staining, we performed additional immunoprecipitation experiments followed by sensitive mass spectrometric (MS) analysis of the precipitates. We have used a variety of strains expressing condensin subunits fused with fluorescent proteins, which were precipitated with the appropriate antibodies (Figure 4A). First, we prepared extracts from early embryos or from ovaries isolated from individuals expressing *Cap-G^FL^-mRFP1* under the control of the genomic regulatory sequences. Like the EGFP-fused Cap-G variant, mRFP1-fused Cap-G is biologically functional as it rescues *Cap-G* mutants to vitality and fertility (data not shown). After immunoprecipitation using anti-mRFP1 antibodies, aliquots of the eluates were separated on an SDS-polyacrylamide gel and stained with silver to visualize the precipitated proteins (Figure 4B). In a parallel experiment, lanes with the eluates were stained with colloidal Coomassie Blue, cut into seven slices each and processed for MS. This procedure allowed a comprehensive evaluation of the proteins associated with the precipitated bait. As a negative control, an extract from *w^1^*-ovaries not containing mRFP1-fused proteins was treated identically. From the list of identified proteins all non-Drosophilid proteins were removed, and then sorted according to the cumulative intensities of the identified peptides. In both the ovary and the embryo extracts, among the top eleven most abundant proteins, SMC2, SMC4, as well as the condensin-I specific subunits Cap-H/Barren and Cap-D2 were identified (Figure 4B). The majority of the peptides specific for SMC2 or SMC4 were detected in gel slices containing proteins of molecular weights corroborating our assignment of the SMCs in the silver stained IP-eluates shown in Figure 2C. However, in the complete list of identified proteins (189 for the embryonic extracts and 537 for the ovary extract), neither Cap-D3 nor Cap-H2 were found, not even represented by a single peptide (Tables S2 and S3). In a complementary approach, we expressed EGFP- and mCherry-fused variants of the condensin II-specific subunit Cap-H2 in ovaries using the GAL4/UAS-system. These variants were shown to be functional as they I) rescue the phenotypic consequences described for Cap-H2 mutants in ovarian nurse cell nuclei and II) trigger a dispersal of polytene chromatin when expressed in the nuclei of larval salivary glands (Figure S9; [24]). Anti-EGFP-Cap-H2 and anti-mCherry-Cap-H2 precipitates from ovarian extracts were separated by SDS-PAGE, stained with colloidal Coomassie Blue, and analyzed by MS (Figure 4C). Within the lists of identified proteins, SMC2 and SMC4 can be found in both experiments. However, the SMCs were ranked much lower in this experiment when compared to the Cap-G immunoprecipitates, indicating that they are of relatively low abundance in the Cap-H2-specific precipitates. Significantly, within the complete list of more than 1200 proteins in both cases, neither Cap-G nor Cap-D3 could be found (Table S4). The N-terminal EGFP- and mCherry-fusions in our Cap-H2 constructs may preclude efficient complex formation. Therefore, we also performed immunoprecipitations of SMC2, from protein extracts of wild type or *SMC2_h_-EGFP* expressing individuals, using either anti-SMC2-antibodies or anti-EGFP antibodies, respectively. In these experiments, we would expect to precipitate both condensin I and condensin II complexes. Again, we could identify the components of the condensin I complex in all cases, but in none of the three experiments, the condensin II-specific components Cap-H2 or Cap-D3 were detected (Figure 4D, Tables S5, S6, S7, S8).

As Cap-G^FL^ is nuclear during interphase, like condensin II subunits in other systems, one might expect condensin II-like phenotypes in *Cap-G* mutant animals rescued by Cap-G^NM^, which is cytoplasmic in interphase. A prominent phenotype in Drosophila *Cap-D3* and *Cap-H2* mutants is the perdurance of nurse cell chromosome polyteny in developing egg chambers [24]. However, in Cap-G^NM^ rescued females, the nurse cell chromosomes disperse on time, arguing against nuclear Cap-G fulfilling a condensin II-like function (compare Figure S10A and S10B). We have ascertained that in the rescue situation in this tissue, Cap-G^NM^ is also excluded from the nuclei (Figure S10C).

Taken together, the phenotypic analysis of nurse cell chromosomes in Cap-G^NM^ rescued females, as well as our immunoprecipitation analyses argue against Cap-G being incorporated into a soluble condensin II-like complex in *Drosophila.* Furthermore our MS results also speak against the presence of soluble condensin II-like complexes in the analyzed extracts in significant amounts.

### Reconstitution of *Drosophila* condensin sub-complexes *in vitro*

The analysis of condensin subunit associations described above involved immunoprecipitations from complexes present in soluble extracts from *Drosophila* tissues. To allow the assessment of direct protein-protein interactions in a more simple system, we analyzed complex formation of various condensin subunits produced in an *in vitro* transcription/translation (IVT) system. In case the molecular mass of the synthesized proteins was sufficiently different, they were co-translated in the presence of [^35^S]methionine, subjected to immunoprecipitation using antibodies against fused epitope-tags, separated by SDS-PAGE, and detected by autoradiography. Otherwise, proteins were translated in different reactions only one of which contained [^35^S]methionine. After mixing the extracts and subsequent immunoprecipitation, the components were detected after SDS-PAGE both by autoradiography and immunoblot.

**Figure 5:**
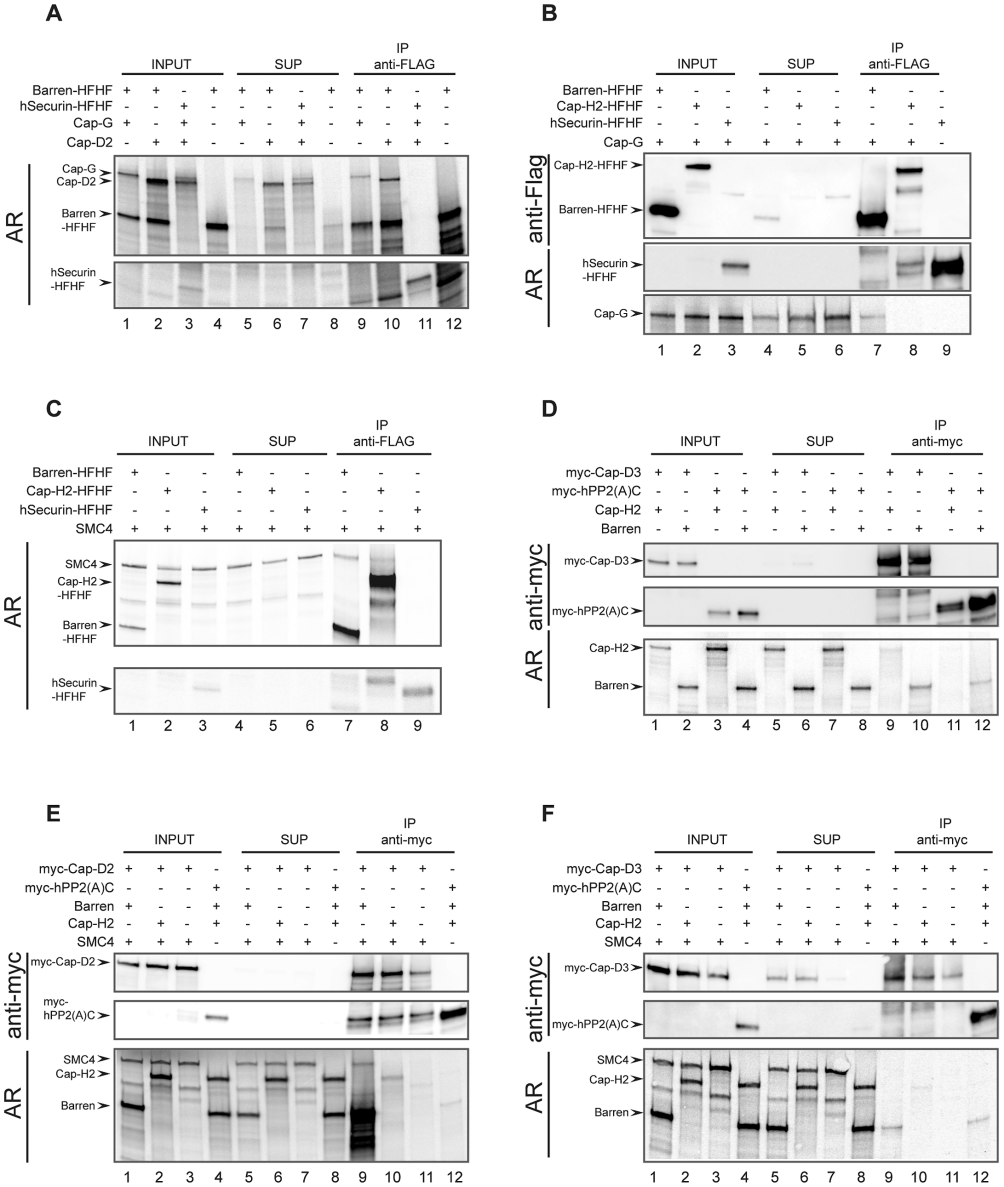
Reconstitution of *Drosophila* condensin sub-complexes *in vitro.* * Drosophila* condensin subunits and control proteins were synthesized by coupled *in vitro* transcription/translation (IVT) either simultaneously in the presence of [^35^S]methionine (A, C) or in separate reactions containing or lacking [^35^S]methionine (B, D, E, F). Hot and cold IVT reactions were mixed (B, D, E, F), or used directly (A, C), and were subjected to immunoprecipitations using anti-Flag (A–C) or anti-myc (D–F) antibody-coated beads. The various combinations of synthesized proteins are indicated by plus signs in the top parts of each panel. Proteins in samples of the input extracts (INPUT), supernatants after precipitation (SUP) and eluates from the antibody-coated beads (IP) were separated by SDS-PAGE. The gels were either dried and directly subjected to autoradiography (AR in (A) and (C)), or they were blotted onto nitrocellulose membranes and the proteins were detected by autoradiography (AR) or immunoblotting using anti-Flag (B) or anti-myc (D–F) antibodies.

To validate our system, we first wanted to demonstrate the physical interactions between the condensin I-specific non-SMC subunits. We used a C-terminally His-FLAG-epitope-tagged Cap-H/Barren (Barren-HFHF) construct as bait. A C-terminally extended Cap-H/Barren variant has been shown to be biologically functional in the fly [28]. As a negative control, we prepared human securin analogously tagged at its C-terminus with His-FLAG (hSecurin-HFHF). Both Cap-G and Cap-D2 can be specifically co-immunoprecipitated with Barren-HFHF, but not with hSecurin-HFHF (Figure 5A). Thus, the *Drosophila* HEAT-repeat containing condensin I subunits interact with the kleisin subunit Cap-H/Barren like their human counterparts [35]. If Cap-G is also part of condensin II, one would expect that it forms a complex with the condensin II-specific kleisin subunit Cap-H2. However, while Cap-G can be readily detected in immunoprecipitates of Barren-HFHF, it is not present in Cap-H2-HFHF immunoprecipitates (Figure 5B). This result once more argues against Cap-G being a condensin II component.

The human kleisin subunits were shown to interact with SMC4 [35]. Consistently, *Drosophila* SMC4 can be precipitated with Barren-HFHF, in low amounts with Cap-H2-HFHF, but not with hSecurin-HFHF (Figure 5C). This result reveals on the one hand a reduced binding efficiency between *Drosophila* Cap-H2 and SMC4, which is consistent with the results from our immunoprecipitation analysis of ovarian extracts containing ectopically expressed Cap-H2-variants (Figure 4C). On the other hand, it demonstrates that in the IVT-system Cap-H2-HFHF is produced in a conformation competent for complex formation, ruling out the possibility that the lack of interaction between Cap-H2-HFHF and Cap-G is due to mis-folded Cap-H2-HFHF. Next we asked whether we could reconstitute the condensin II-specific interaction between Cap-D3 and Cap-H2. To this end, we synthesized a Cap-D3 variant fused at its N-terminus with six copies of the human c-myc-epitope (myc-Cap-D3). In these experiments, we used as negative control the catalytic (C)-subunit of human protein phosphatase 2A, also with an N-terminal myc_6_-tag (myc-hPP2(A)C). Cap-H2 could be identified in myc-Cap-D3 immunoprecipitates, but not in myc-hPP2(A)C precipitates (Figure 5D). However, the co-precipitation efficiency was again very low. Cap-H/Barren was also detected in myc-Cap-D3 immunoprecipitates, but this protein was also precipitated by myc-hPP2(A)C, arguing for non-specific associations. To underscore the biological relevance of these *in vitro* studies, we attempted to form ternary complexes. Based on the geometry of the human condensin complexes, Cap-D2 does not directly interact with the SMC subunits, but the kleisin subunit Cap-H/Barren is expected to bridge Cap-D2 and SMC4. Indeed, SMC4 can be precipitated together with myc-Cap-D2 when Cap-H/Barren is present, but not in its absence (Figure 5E, compare lanes 9 and 11). When Cap-H2 was included in an analogous reaction instead of Cap-H/Barren, Cap-H2 was precipitated with low efficiency, but SMC4 could not be detected (Figure 5E, lane 10). In an effort to reconstitute an analogous condensin II subcomplex, we precipitated myc-Cap-D3 in the presence of both Cap-H2 and SMC4 or just SMC4. In this case, no ternary complex could be detected and only inefficient co-precipitation of Cap-H2 with myc-Cap-D3 was observed (Figure 5F, lane 10). Cap-H/Barren did not co-precipitate with Cap-D3 above background. Taken together, our *in vitro* complex forming studies confirm the predicted interactions among the *Drosophila* condensin I-specific subunits. However, the complex forming potential between condensin II-specific subunits is limited and we find again no evidence for incorporation of Cap-G in a condensin II-like subcomplex.

## Discussion

We set out to test the hypothesis that in *Drosophila*, Cap-G might be part of both condensin I and condensin II. This hypothesis is based on the facts that i) no condensin II-specific Cap-G2 homolog can be identified in the *Drosophila* genome and ii) that SMC2 and SMC4 are also part of both condensin complexes.

The localization pattern of Cap-G-EGFP in interphase initially suggested its participation in a condensin II-like complex since it was found to be nuclear like vertebrate condensin II subunits [9]–[11]. At least, a functional importance was suggested by the preferential nuclear localization of Cap-G and its different dynamics in chromatin association when compared to the other EGFP-fused condensin I subunits. However, the intriguing observation that flies are viable and fertile, when they exclusively express a C-terminal truncation variant of Cap-G, which is nuclear excluded in interphase and gains access to chromatin only around NEBD, suggests that its nuclear localization is dispensable for proliferation and development, at least under laboratory conditions. Furthermore, the observed heterochromatic enrichment of Cap-G and its initiation of loading at the centromeric regions are obviously not essential. It is possible that the Cap-G C-terminus, which contains many predicted phosphorylation sites in *Drosophila* and other organisms [36] may fine tune Cap-G activity. This fine-tuning is probably required for the restoration of full fertility in both sexes and early syncytial development, as shown by the defects when no full length Cap-G is provided by the mother. In this respect, the C-terminus might be required for full length Cap-G to be sequestered into the nucleus to avoid any dominant negative effects in the cytoplasm.

SMC2_h_-EGFP and EGFP-Cap-D2 localize like Cap-H/Barren-EGFP [28] in the cytoplasm during interphase and rapidly associate with chromatin during early stages of mitosis. Intriguingly, these subunits associate significantly later with chromatin than Cap-G-EGFP, indicating that Cap-G has the potential to bind to chromatin in the absence of the other condensin subunits. This notion is supported by the observation that Cap-G^C^ can associate with chromatin in late anaphase, at a time point when the other subunits dissociate. Recently, it has been shown in human tissue culture cells and fission yeast that Cap-H binds to the N-terminal tail of histone 2A and the variant histone 2A.Z. *In vitro* studies have revealed that this binding can occur independent of other condensin subunits [37]. While these results are consistent with chromatin targeting of condensin via Cap-H in these systems, our findings suggest that in *Drosophila*, Cap-G may direct chromatin targeting of condensin. The target molecule on chromatin, which is recognized by *Drosophila* Cap-G, remains to be identified.

While our study is the first report on the dynamics of SMC2 localization in *Drosophila* during the cell cycle, our data on Cap-D2 appear to be at odds with studies on fixed S2 tissue culture cells using anti-Cap-D2-antibodies [18]. In this study, Cap-D2 was reported to be primarily nuclear. This discrepancy can be explained by the different tissues analyzed. Nuclear import may be slow for Cap-D2, as, in fact, Savvidou et al. [18] observe increasing nuclear concentration of Cap-D2 when the cells progress through G1-S-G2. During the rapid syncytial divisions, nuclear import of Cap-D2 may not be efficient. Analysis of other tissues of EGFP-Cap-D2 expressing animals indeed showed nuclear localization, for example in ovarian follicle cells (data not shown). Interestingly, nuclear localization of Cap-H2 has also been described to progressively increase in more advanced ovarian nurse cell nuclei when compared with nuclei at younger stages [24], own unpublished observation).

Condensin complexes have been initially identified and characterized in the biochemically tractable *Xenopus* egg extract system [4]. In mitotic extracts, soluble 13S heteropentameric holocomplexes as well as 8S SMC2/SMC4 dimers were readily detected. Besides this initial identification of the complex later termed condensin I, condensin II was also detected in high-speed supernatants of *Xenopus* egg extracts [12], as well as in HeLa cell lysates [12], [38]. Quantification revealed that in the *Xenopus* egg extract system condensin I is present in roughly five-fold excess over condensin II while in HeLa cells both complexes occur in approximately equimolar amounts [12]. These differences in abundance are paralleled by a different appearance of condensed chromosomes. While in HeLa cells, metaphase chromosomes appear short and thick, the condensed chromosomes in the *Xenopus* egg extract system are rather long and thin. Intriguingly, experimentally shifting the ratio of condensin I∶condensin II in *Xenopus* egg extracts from ∼5∶1 to ∼1∶1 resulted in shorter and thicker chromosomes [31]. As metaphase chromosomes in *Drosophila* are also short and thick, one would expect a roughly balanced abundance of the two condensin complexes, if condensin I and II play comparable roles in the fly. As we did not detect any soluble endogenous condensin II complexes in our immunoprecipitation analyses, this is apparently not the case. We have analyzed extracts from ovaries and embryos. *Cap-H2* mutants display a phenotype in ovarian nurse cell nuclei suggesting that *Cap-H2* is expressed at this stage [24]. Also, the temporal expression data provided by the modENCODE project reveal expression of both *Cap-H2* and *Cap-D3* in ovaries and in early embryos, albeit at only low to moderate levels [39]. In fact, these levels are significantly lower than those reported for condensin I-specific subunits in most tissues indicating that condensin II-like complexes must be of low abundance. Our analysis of ovarian extracts derived from females overexpressing Cap-H2-fusion proteins circumvented the issue of low endogenous expression levels. Indeed, in these experiments, SMC2 and SMC4 were found to be associated with overexpressed Cap-H2, but peptide intensities and unique peptide numbers were significantly lower than in the experiment, in which proteins in association with Cap-G-mRFP1 in ovaries were assessed. Also, as our *in vitro* interaction assays revealed only weak affinities of Cap-H2 towards Cap-D3 and SMC4 in solution, a condensin II-like holocomplex in *Drosophila* may be functionally assembled in an efficient manner only on chromatin, unlike the situation found in vertebrates. Published studies on the phenotypic consequences of the loss of *Cap-D3* or *Cap-H2* have shown that these phenotypes can be modified by mutations in other condensin subunit genes (namely *Cap-H2*, *Cap-D3* and *SMC4*), thus revealing genetic interactions [23], [24], [40]. However, it remains to be shown, whether these genetic interactions are based on a physical interaction of these subunits bound to the chromatin. Furthermore, such a chromatin-associated condensin II-like holocomplex is unlikely to play a mitotic role, given the absence of mitotic phenotypes in *Cap-H2* and *Cap-D3* mutants [18], [23], [24], which is also consistent with the failure of EGFP-Cap-H2 to load onto mitotic chromatin (data not shown).

Cap-G was not found in association with overexpressed Cap-H2, even though Cap-G would be expected to bind to the kleisin component if it was part of a condensin II-like complex [35]. The direct binding assays of *in vitro* translated proteins also did not produce any indication of an association of Cap-G with Cap-H2, rendering the proposal of the participation of Cap-G in a condensin II-like complex highly unlikely. So the question remains whether a second HEAT-repeat containing protein besides Cap-D3 is part of a putative condensin II complex in *Drosophila*. BLAST analyses do not produce Cap-G2 homologs encoded in the *D. melanogaster* genome or in any of the sequenced genomes of dipterans. It is possible that a Cap-G2 homolog does exist in *Drosophila*, but has escaped detection using the BLAST algorithms because it might have diverged significantly during evolution. Therefore, we have scrutinized the list of proteins identified in the Cap-H2 immunoprecipitates for possible Cap-G2 candidates by the virtue of a size above 100 kDa, and an extended stretch of predicted HEAT repeats in the N-terminal region, but with dissimilarity to importins/exportins which also have blocks of HEAT repeats in their N-termini. However, none of the proteins contained in the list of immunoprecipitated proteins qualifies as a Cap-G2 homolog based on these criteria (data not shown). Thus, the possibility remains that condensin II has diverged in dipterans to function as a mainly chromatin-bound heterotetrameric complex lacking a Cap-G2 subunit. Moreover, in combination with the facts that *Cap-H2* and *Cap-D3* loss-of-function mutants have no obvious mitotic phenotype [18], [23] and that these two subunits have been shown to participate in such diverse processes as transvection, the regulation of AMP-expression or chromosome territory formation [23]–[25], [40], our results support a model in which a *Drosophila* condensin II-like complex has functionally specialized beyond regulation of chromatin structure during nuclear divisions.

## Materials and Methods

### *Drosophila* stocks

Fly stocks were obtained from the Bloomington *Drosophila* Stock Center at Indiana University, unless indicated otherwise. Expression constructs for condensin subunits were generated by cloning genomic fragments isolated from bacterial artificial chromosomes (BACs) obtained from CHORI BacPac Resources into appropriate vectors, or cDNAs obtained from the *Drosophila* Genomic Resource Center (DGRC) into the vectors pUAST or pUASP1 [14], [41]. Appropriate restriction sites for cloning were introduced by PCR with primers containing the recognition sequences for the respective enzymes. The integrity of coding regions amplified by PCR was verified by subsequent DNA sequence analysis. Transgenic flies were generated by using established germ line transformation protocols for microinjection into *w^1^* embryos (pUAST, pUASP1 and pBac-constructs) or into embryos expressing the PhiC31 integrase and containing an attP landing site at specific genomic sites [42].

For the construction of fly stocks expressing an EGFP-fused variant of SMC2, a 5.2 kb fragment containing *SMC2* including its flanking genomic regions was amplified from the BAC clone CH321-59P12 as template and cloned into the pattB vector [42]. A 1370 bp internal *Pst*I/*Mlu*I *SMC2*-fragment was subcloned into the *p*SLfa1180fa vector [43] and fused with the EGFP-coding sequence using a *Bsp*EI site introduced by inverse PCR. The EGFP tag was fused internally between amino acid residues G582 and S583 of SMC2 within the hinge region (SMC2_h_-EGFP). Internal fusions within the hinge region of yeast SMC1 and SMC3 have been shown to be functionally tolerated [44]. The modified fragment was cloned back into the pattB-SMC2 vector. Transgenic flies were generated via injection of the pattB-SMC2_h_-EGFP plasmid into *y^1^, w^1^, M[vas-int]ZH2A; M[3x3P-RFP,attP′]ZH96E* embryos [42].

For the construction of fly stocks expressing an EGFP-fused variant of Cap-D2 under control of the genomic regulatory sequences, a 6.8 kb genomic fragment encompassing Cap-D2 and 600 bp upstream of the transcriptional start site as well as 1,600 bp downstream of the poly(A) site was cloned via recombineering [45] into pattB using the BAC CH321-26K05 as sequence source. A 1.5 kb *Not*I/*Acc*65I fragment of the 5′-terminal Cap-D2 region was isolated from pattB-Cap-D2 and subcloned into the pBluescriptSK vector (Stratagene). The naturally occurring *Nco*I site at the Cap-D2 translational initiation codon was used to insert a PCR-amplified fragment encoding EGFP, flanked by *Pci*I sites, which are compatible with *Nco*I. The 2.2 kb *EGFP*-fused *Not*I/*Acc*65I 5′-terminal Cap-D2 fragment was cloned back into the *Not*I/*Acc*65I cleaved pattB-Cap-D2. Transgenic flies were generated via injection of the pattB-EGFP-Cap-D2 plasmid into *y^1^, w^1^, M[vas-int]ZH2A; M[3x3P-RFP,attP′]ZH22A* embryos [42].

For the construction of fly stocks expressing EGFP- and mRFP1-fused variants of Cap-G under control of the genomic regulatory sequences, a 1.2 kb *Xho*I fragment encompassing the 3′-terminal region of the *Cap-G* reading frame and downstream regulatory sequences was cloned from a genomic *Cap-G* pBac rescue construct [14] into the vector pLitmus 28 (New England Biolabs). After introduction of a *Not*I restriction site immediately upstream of the translational stop codon by inverse PCR, PCR-amplified fragments encoding either EGFP or mRFP1 flanked by *Not*I sites were cloned into this newly generated site. The modified 1.9 kb *Xho*I fragments were excised from the pLitmus 28 constructs and cloned back into the pBac Cap-G rescue constructs. Transgenic flies were generated via injection of the pBac-Cap-G-mRFP1 and pBac-Cap-G-EGFP plasmids into *w^1^* embryos using established procedures [43]. The genomic region encoding Cap-G-EGFP was also cloned into the pattB vector and transgenic lines were established after injection into *y^1^, w^1^, M[vas-int]ZH2A; M[3x3P-RFP,attP′]ZH96E* embryos.

For the construction of *pUAST-Cap-G-EGFP* vectors containing various *Cap-G* fragments, the corresponding *Cap-G* coding regions were PCR-amplified from the cDNA clone *SD10043* and cloned into *pUAST-MCS-EGFP* [46]. Fragments encoding the following Cap-G-variants were amplified: Cap-G^FL^ (full length, aa 1–1351); Cap-G^NM^ (aa 1- 977); Cap-G^NM1^ (aa 1–848); Cap-G^NM4^ (aa 243- 977); Cap-G^C^ (aa 958–1351). For the construction of *pUASP1-Cap-G^NM^-EGFP*, the *Cap-G^NM^-EGFP-*fragment was transferred from *pUAST-Cap-G^NM^-EGFP* into *pUASP1* [14]. The constructs were used for P-element-mediated germ line transformation by injection into *w^1^* embryos following established procedures. For all experiments, the following established lines were used: *UAST-Cap-G^FL^-EGFP II.2, UAST-Cap-G^FL^-EGFP III.2, UAST-Cap-G^NM^-EGFP III.2, UAST-Cap-G^C^-EGFP II.3, UAST- Cap-G^C^ -EGFP III.2, UAST-Cap-G^NM1^-EGFP II.1, UAST-Cap-G^NM4^-EGFP II.1, UASP1-Cap-G^NM^-EGFP III.4, UASP1-Cap-G^NM^ III.2. Cap-G^NM^ –EGFP* and *Cap-G^NM^* were also cloned into the pattB vector containing the flanking *Cap-G* genomic regulatory elements ensuring expression at physiological levels. Transgenic lines were established after injection into *y^1^, w^1^, M[vas-int]ZH2A; M[3x3P-RFP,attP′]ZH96E* embryos.

For the construction of *pUASP1-EGFP-Cap-H2* and *pUASP1-mCherry-Cap-H2*, the *Cap-H2* coding region (based on the *Cap-H2-RE* annotation) was isolated using *NcoI/XhoI* from the cDNA clone SD18322 and subcloned into pLitmus28. The resulting plasmid pLitmus28-Cap-H2 was cleaved with *Avr*II/*Nco*I and PCR-fragments encoding mCherry and EGFP were inserted as *AvrII/PagI* fragments. The *EGFP-Cap-H2* and *mCherry-Cap-H2* cassettes were finally transferred as *SpeI/Asp718-*fragments into *pUASP1* to generate *pUASP1-EGFP-Cap-H2* and *pUASP1-mCherry-Cap-H2*, respectively, which were used for P-element-mediated germ line transformation. For all experiments, the transgene insertions *UASP1-EGFP-Cap-H2 II.4* and *UASP1-mCherry-Cap-H2 II.1* were used.

For expression of UAS-transgenes, we used *da-GAL4 G32* [47], *F4-GAL4* [48], maternal *α4tub-GAL4-VP16* [49] and *tubP-GAL4*.

Rescue experiments were performed using *trans*-heterozygous mutant allele combinations of the respective genes, simultaneously expressing our transgenes either under control of the flanking genomic regulatory regions or under UAS-control driven by the ubiquitous active *GAL4*-driver *da-GAL4 G32* or by *α4tub-GAL4-VP16* in the case of *Cap-H2*. The following alleles were used: *Cap-G^1^* and *Cap-G^6^* [14], *Cap-D2^f03381^*, *Cap-D2 ^Df(3R)01215^, SMC2^jsl2^, SMC2^f06842^, SMC2^Df(2R)BSC429^*, *Cap-H2^Df(3R)Exel6159^* , *Cap-H2^EY09979^* and *Cap-H2^TH2^* [24]. For *Cap-G*, *Cap-D2* and *SMC2*, complementation of the lethality associated with the *trans*-heterozygous mutant situation was assessed. For *Cap-G*, rescued *trans*-heterozygous individuals could be readily identified by the recessive markers *al*, *b*, *c* and *sp* present on the *Cap-G^1^* and *Cap-G^6^* chromosomes [14]. For *Cap-H2*, suppression of the delayed dispersal of nurse cell chromatin observed in *Cap-H2* mutant ovarioles [24] was monitored upon transgene expression. Furthermore, the phenotype upon overexpression of *EGFP-Cap-H2* and *mCherry-Cap-H2* in larval salivary glands was compared with the phenotype obtained after the GAL4 dependent *Cap-H2* overexpression using the allele *Cap-H2^EY09979^*, which is an UAS containing P-element inserted upstream of *Cap-H2*.

To drive expression of *Cap-G^NM1^-EGFP* or *Cap-G^NM4^-EGFP* together with *His2Av-mRFP1*, individuals of the corresponding UAS-lines were crossed with w*, α-tub-GAL4-VP16, gHis2Av-mRFP1 II.2 flies (generously provided by C. Lehner, University of Zurich).

To express *HP1-mRFP1* together with *Cap-G^FL^-EGFP* or *Cap-G^C^-EGFP*, we generated recombinant chromosomes containing either *UAST-Cap-G^FL^-EGFP II.2* or *UAST-Cap-G^C^-EGFP II.3* together with *gmRFP1-HP1 II.1* [50] using standard genetic techniques.

To co-express *Cap-G^FL^-EGFP* with *Cid-mRFP1*, both under control of the flanking genomic sequences, lines were generated by classical genetic techniques containing the *gCap-G^FL^-EGFP III.1* and *gCid-mRFPII.1* [28] transgenes.

For chromatin loading analyses, chromosomes carrying a transgene allowing expression of His2Av fused with mRFP1 [51] were combined with *gCap-G-EGFP III.1*, or *gSMC2_h_-EGFP^ΦX-96E^* or *gCap-D2-EGFP^ΦX-22A^*.

### Antibodies

Antibodies against the human c-myc epitope [52], *Drosophila* Cap-H/Barren [22] and *Drosophila* Cap-D2 [18] have been described previously.

Rabbit-anti-Flag (Sigma), mouse-anti α-Tubulin (Sigma) as well as secondary antibodies (Jackson laboratories) were obtained commercially. Antibodies against EGFP and mRFP1 were raised in rabbits using bacterially expressed full length proteins as antigen. The anti-mRFP1 antibodies also recognize and precipitate mCherry-fused proteins. Mouse monoclonal antibodies against EGFP were purchased from Roche Biochemicals or were a gift from D. van Essen and S. Saccani (MPI Freiburg, Germany).

Antibodies against Cap-G and SMC2 were raised in rabbits using bacterially expressed N-terminal protein fragments of Cap-G (aa 1- 553) and SMC2 (aa 1–313), respectively. The antisera were affinity purified using standard procedures [53]. For immunoblotting, the antibodies were used at a 1∶3000 dilution. A mouse monoclonal antibody directed against HP1 was obtained from the Developmental Studies Hybridoma Bank (clone C1A9; dilution 1∶1000 for immunoblotting).

### Microscopy and image processing

For *in vivo* microscopy, embryos at the desired developmental stage were collected and processed as previously described [54]. Single-stack confocal images were acquired every 18 or 20 sec using a Leica SP5 confocal microscope (Leica Microsystems, Germany), equipped with a 63× oil-immersion objective, a 458–514 nm Ar laser and a 561 nm DPSS laser for the excitation of EGFP and mRFP1, respectively. For fixed samples stained with Hoechst 33258, a 405 nm UV- diode laser was used in addition, and confocal images were acquired with a 40× oil-immersion objective.

Images were processed using ImageJ 1.46 (National Institute of Health, USA) and Adobe Photoshop CS4 (Adobe Systems Inc.). In some images, shot noise was decreased with a Gaussian filter.

Quantitative fluorescence measurements to determine chromatin association of the EGFP-fused condensin subunits was done as described in [28] with the exception that a Leica SP5 confocal system was used for analysis of EGFP-Cap-D2 and SMC2_h_-EGFP. The analyzed genotypes were *gCap-G-EGFP III.1, gHis2Av-mRFP1 III.1/TM3, Ser* or *gHis2Av-mRFP1 II.2; gSMC2_h_-EGFP^ΦX-96E^* or *gCap-D2-EGFP^ΦX-22A^; gHis2Av-mRFP1 III.1*. or *SMC2^f06842^/SMC2^Df(2R)BSC429^*; *gSMC2_h_-EGFP^ΦX-96E^* .To quantify Cap-G-EGFP in centromeric regions, embryos co-expressing *Cap-G-EGFP* and *Cid-mRFP* were analyzed by laser scanning time lapse microscopy while progressing through the syncytial cycle 12. Small circular regions of interest (R.O.I.s) were defined in the channel for Cid-mRFP fluorescence, one encircling a centromere (cen-proximal) and one of the same size in a region within the nucleus but not encircling a centromere. The identical R.O.I.s were applied to the channel for Cap-G-EGFP fluorescence and the ratio of the cen-proximal fluorescence intensity:cen-distal fluorescence intensity was calculated. For each time point, 62 pairs of R.O.I.s from three different embryos were evaluated.

### Immunoblotting and immunoprecipitation experiments

For immunoblotting experiments, ovaries of 4–5 days old females were dissected in 1× PBS and homogenized in sodium dodecyl sulfate polyacrylamide gel electrophoresis (SDS-PAGE) sample buffer. Protein samples corresponding to 5 ovaries were loaded on Tris-glycine based polyacrylamide gels and blotted onto nitrocellulose membranes.

For the immunoprecipitation experiments, 5–8 h old embryos expressing fluorescently tagged *Cap-G* variants were collected on apple-juice agar plates and dechorionized. Alternatively, we dissected ovaries in 1×PBS from females expressing epitope-tagged condensin subunits. These tissues (150 µl embryos or 300 ovaries) were homogenized in 4 volumes of lysis buffer (50 mM HEPES at pH 7.5, 60 mM NaCl, 3 mM MgCl_2_, 1 mM CaCl_2_, 0.2% Triton X-100, 0.2% Nonidet NP-40, 10% glycerol) including protease inhibitors (2 mM Pefabloc, 2 mM Benzamidin, 10 µg/ml Aprotinin, 2 µg/ml Pepstatin, A, 10 µg/ml Leupeptin). In the experiment shown in Figure S6, aliquots of the raw extracts were treated with a mixture of DNaseI and nuclease S7 for 45 min at 4°C to solubilize chromatin. The extracts were cleared by centrifugation (20 min, 14000×g; 4°C) and 200–400 µl of the supernatants were used for immunoprecipitation with anti-EGFP-, anti-mRFP1-, or anti-SMC2-antibodies bound and covalently cross-linked using dimethyl pimelimidate to Protein A-Sepharose (Affi-Prep Protein A, BIORAD; 25 µg of affinity purified antibodies bound to 30 µl of Protein A-Sepharose slurry). In the experiment shown in Figure S6, mouse monoclonal antibodies (Roche) were coupled to Protein G-Sepharose (GE Healthcare). After 3–4 h incubation at 4°C with gentle agitation, the Sepharose was washed for four times with 1 ml of lysis buffer. Bound polypeptides were eluted by incubation with 40 µl of elution buffer (50 mM Tris/HCl at pH 6.8; 2% (w/v) SDS) for 10 min at 37°C and/or by addition of 40 µl SDS-PAGE sample buffer and subsequent incubation at 95°C for 5 min (“hot elution”).

The immunoprecipitates were subjected to SDS–PAGE followed by silver staining (“PageSilver Silver Staining Kit”, Fermentas) or by western blot analysis.

### Mass spectrometric analysis

For mass spectrometric analysis, immunoprecipitates were separated by SDS-PAGE on precast gradient gels (Serva, Heidelberg) and the proteins were visualized by staining with colloidal Coomassie Blue according to [55]. Entire gel lanes containing immunoprecipitates were cut into slices. Proteins were extracted from the gel pieces, digested with trypsin, separated via on-line nanoLC and analyzed by electrospray tandem mass spectrometry at an LTQ Orbitrap mass spectrometer. The complete lists with the identified proteins are available in the supplementary information.

### *In vitro* transcription/translation reactions

DNA fragments encoding different regions of the condensin subunits were amplified by PCR and inserted into the vectors *pCS2(F/A)*, *pCS2(F/A)-HFHF* (allowing a fusion of a C-terminal His_6_ Flag His_6_ Flag epitope tag), and/or *pCS2-myc_6_(F/A)* (allowing a fusion of an N-terminal myc_6_-epitope tag) , which all contain *FseI/AscI*-restriction sites within their MCS. Condensin coding regions were amplified from the cDNA clones *SD10043* (Cap-G), *LD40412* (Cap-D2), *RE48802* (Cap-H/Barren), *SD18322* (Cap-H2, based on the *Cap-H2-RE* annotation) and *RE74832* (Cap-D3, based on the *Cap-D3-RA* annotation).

To generate *pCS2-Cap-G-EGFP*, the *Cap-G-EGFP* fragment was transferred from *UAST-Cap-G^FL^-EGFP* into *pCS2(F/A)*. To generate *pCS2-SMC4*, the corresponding coding region was amplified using first strand cDNA derived from reverse transcription of mRNA extracted from *w^1^*-embryos, using the “RNeasy Mini Kit” and the “Omniscript RT Kit” (Qiagen), and inserted into *pCS2(F/A)*.

For controls, the plasmids *pCS2-hSecurin-HFHF* and *pCS2-myc6-hPP2A(C)* (generously provided by O. Stemmann) were used, which contain the coding DNA sequences for human securin and the catalytic subunit of the human protein phosphatase 2A, respectively.

Coupled *in vitro* transcription/translation reactions (IVT) were performed using the “TNT SP6 Coupled Reticulocyte Lysate System” or the “TNT SP6 Quick Coupled Transcription/Translation System” (Promega) according to the manufacturer's instructions. Up to 3 different plasmids (final amount of 2 µg DNA total) were included in 25 µl reaction mixtures. For radioactive labeling, 0.4 µM [^35^S]methionine (1000 Ci/mmol) was added to the reaction mix. In some instances, the produced proteins migrated at almost the same position during SDS-PAGE. In these cases, only the components without an epitope tag were translated in the presence of [^35^S]methionine. The epitope tagged variants were translated in a separate reaction in the absence of radioactive label. Afterwards, the reactions were mixed and subjected to immunoprecipitation using 5 µl of mouse-anti-Flag-Agarose-slurry (Sigma, A1080) or 5 µl Protein-A-Sepharose beads to which monoclonal mouse antibodies against the myc-epitope had been covalently crosslinked with dimethyl pimelimidate [53]. After 3 h incubation at 4°C with gentle agitation and a subsequent brief centrifugation, the supernatants were removed and immunoprecipitates were washed 3 times with 1 ml of lysis buffer. Bound polypeptides were eluted by addition of 40 µl SDS-PAGE sample buffer and subsequent incubation at 95°C for 5 min. Precipitated polypeptides as well as samples derived from the input and supernatant fractions were resolved by SDS-PAGE and analyzed by immunoblotting and/or autoradiography (FLA 7000 Phosphoimager, Fuji Corp.)

## Supporting Information

Figure S1 Construction and expression of EGFP-fused condensin variants. (A) Schematic presentation of all constructs, which are based on genomic DNA sequences. Orange bars represent the condensin reading frames including introns and the green bars the fused *EGFP* reading frame. Light grey bars indicate 5′- and 3′-UTRs. Dark grey bars represent 5′- and 3′-flanking genomic regions. While Cap-G and Cap-D2 were tagged at their C-terminus and N-terminus, respectively, the *EGFP* reading frame was inserted into *SMC2* between the codons for amino acids glycine 582 and serine 583. (B) Analysis of expression levels. Extracts were prepared from 0–3 hrs old embryos derived from mothers carrying one transgene copy and which had been mated with wild type (*w^1^*) males. Proteins contained in four serial dilutions of each extract were separated by SDS-PAGE, blotted, and detected with monoclonal anti-EGFP antibodies (αEGFP) and anti-α-tubulin antibodies (αtubulin) as loading control.
(TIF)

Figure S2 Dynamics of chromatin association of SMC2_h_-EGFP in an *SMC2* mutant background. (A) The fluorescence intensity of SMC2_h_-EGFP in syncytial embryos laid by mothers with the genotype *SMC2^f06842^/SMC2^Df(2R)BSC429^*; *gSMC2_h_-EGFP^ΦX-96E^* was determined for selected nuclei progressing through mitosis 12 in each frame, and is plotted as relative intensity per nucleus (green curve, SMC2 rescue). As these embryos did not contain the red fluorescent His2Av-mRFP1, a correction for chromatin compaction (as done in Figure 1B) was not possible. Thus, the data for SMC2_h_-EGFP in a *SMC2^+^*-background was processed analogously (red curve, SMC2 (w/o HismRFP)). Data series were aligned accordingly to anaphase onset (t_0_ = last metaphase frame). Data sets from a total of 28 nuclei from 12 embryos were aligned. The curves for Cap-D2, Cap-H/Barren, Cap-G, and SMC2 are the same as in Figure 1B and are shown for reference. The times of initiation of chromatin condensation (ICC) and NEBD are indicated by the dotted and dashed red lines, respectively. (B) Western blot analysis of extracts from 0–3 hrs old embryos with the genotype y^1^, M{vas-int.Dm}ZH-2A, w*; M{3xP3-RFP.attP′}ZH-96E (ΦX 96E) or laid by mothers with the genotype *SMC2^f06842^/SMC2^Df(2R)BSC429^*; *gSMC2_h_-EGFP^ΦX-96E^* (SMC2 rescue). The blot was probed with anti-SMC2 antibodies (upper panel) and anti-α-tubulin antibodies as loading control (bottom panel).
(TIF)

Figure S3 Time-lapse analysis of Cap-G^NM^-EGFP association with chromatin. Subcellular localization and chromatin association of Cap-G^NM^-EGFP observed in a living embryo progressing through epidermal mitosis 14. Expression of the *UAST-Cap-G^NM^-EGFP* transgene was driven by *α4-tub-GAL4-VP16*. NEBD occurs between time points −4∶00 and −3∶40 as indicated by the influx of Cap-G^NM^-EGFP into the nuclear space. Chromatin enrichment of Cap-G^NM^-EGFP is detectable starting from time point -3∶20. Individual frames of time lapse movies are shown with time points indicated in min∶sec (t = 0, anaphase onset). In the merged panels, His2Av-mRFP and Cap-G^NM^-EGFP are shown in red and green, respectively. Scale bar is 5 µm.
(TIF)

Figure S4 Western blot analysis of proteins associated with Cap-G fragments. Extracts from 3–6 h old embryos expressing various EGFP-fused Cap-G-fragments driven by *α4-tub-GAL4-VP16* were subjected to immunoprecipitation with rabbit-anti-EGFP antibodies. Bound proteins were eluted in two steps with increasing stringency. Precipitates were separated by SDS-PAGE and blotted onto a nitrocellulose membrane. The blot was probed with antibodies against Cap-D2 (top panel), against Cap-H/Barren (middle panel) and against EGFP (lower panel). Cap-D2 and Cap-H/Barren were efficiently precipitated by Cap-G^FL^-EGFP (FL) and Cap-G^NM^-EGFP (NM) and were eluted during the first step (IP 1^st^ elution), while they were much less efficiently precipitated by Cap-G^NM4^-EGFP (NM4) and Cap-G^C^-EGFP (C). The second elution step (IP 2^nd^ elution) mainly reveals the recovery of the EGFP-fused Cap-G-fragments (indicated by arrowheads). Note that Cap-G^NM4^-EGFP and Cap-G^C^-EGFP migrate at the same position in the SDS-polyacrylamide gel. Asterisks indicate proteins cross-reacting with the anti-EGFP antibody.
(TIF)

Figure S5 Subcellular localization of Cap-G^NM1^-EGFP and Cap-G^NM4^-EGFP. Living embryos expressing *His2Av-mRFP1* and *Cap-G^NM1^-EGFP* (A) or *Cap-G^NM4^-EGFP* (B) were observed while progressing through epidermal mitosis 14. Individual frames of time lapse movies are shown with time points indicated in min∶sec. The top rows show the distribution of the EGFP-fused Cap-G fragments and the bottom rows of His2Av-mRFP1. Red triangles highlight individual cells progressing through mitosis. Scale bar is 5 µm.
(TIF)

Figure S6 HP1 does not co-precipitate with Cap-G. Extracts of 4–7 hrs old embryos expressing *UAS-Cap-G^FL^-EGFP* or *UAS-Cap-G^C^-EGFP* driven by *da-GAL4* were either treated (+) or not treated (−) with nuclease to solubilize chromatin. After centrifugation to pellet debris and undigested chromatin, the supernatant was used for immunoprecipitation with monoclonal anti-EGFP antibodies. Proteins in the raw extracts, the cleared supernatants (INPUT) and the eluates after immunoprecipitation (ELUATE IP αEGFP) were separated by SDS-PAGE, blotted, and detected using a different anti-EGFP antibody, anti-SMC2, and anti-HP1 antibodies. Asterisks denote cross-reactions of the HP1-antibody. HP1 is not detectable in the IP eluates, while SMC2 is readily and specifically co-precipitated with Cap-G^FL^. Note that in the INPUT samples treated with nuclease, the abundance of HP1 is increased when compared with the samples without nuclease treatment, indicating successful chromatin solubilization.
(TIF)

Figure S7 Quantitative measurement of Cap-G-EGFP accumulation at centromeric regions. Embryos co-expressing *Cap-G^FL^-EGFP* and *Cid-mRFP1* were observed while progressing through syncytial mitosis 12. (A) Graphic representation of the ratios between mean Cap-G-EGFP fluorescence intensity of centromere proximal regions (MFI cen-proximal) and the mean Cap-G-EGFP fluorescence intensity of centromere distal regions (MFI cen-distal) plotted over time. t = 0 min corresponds to anaphase onset. n = 62 for each time point. ICC (initiation of chromosome condensation) and NEBD (nuclear envelope breakdown) time points are adapted from the experiment shown in Figure 1. (B) Example of images illustrating the selection of R.O.I.s for the quantitative fluorescence measurements.
(TIF)

Figure S8 *Cap-G* mutant animals rescued by Cap-G^NM^-EGFP: Lack of endogenous *Cap-G* expression and failure of Cap-G^NM^-EGFP to localize to prophase chromatin. (A) Extracts were prepared from ovaries from animals expressing wild type *Cap-G* (*w^1^*, lane 1) or the C-terminally truncated variant *Cap-G^NM^-EGFP* in a *Cap-G^1^/Cap-G^6^ trans*-heterozygous mutant background (lanes 2 and 3) under control of the ubiquitous driver *da-GAL4*. The transgenes were contained either in the vector pUAST (lane 2) or in the vector pUASP1 (lane 3). Extracts corresponding to 5 ovaries were separated on an SDS-polyacrylamide gel, blotted onto a nitrocellulose membrane and the blot was probed with anti-Cap-G antibodies recognizing the N-terminus of the protein (anti-Cap-G(N)) or as a loading control with anti-tubulin antibodies. Note that full-length Cap-G is partially degraded in lane 1. (B) Embryos derived from mothers with the genotype *Cap-G^1^/Cap-G^6^*; *da-GAL4/UASP1-Cap-G^NM^-EGFP III.4* were fixed and treated with Hoechst 33258 to stain the DNA. In the left panel, nuclei in the periphery of an embryo are shown progressing through prophase of syncytial mitosis 13. The right panel shows epidermal nuclei of an embryo progressing through mitosis 14. Cells in prophase (Pro), metaphase (Meta) and anaphase (Ana) are indicated by yellow arrowheads. Prophase cells were identified by the appearance of condensed chromatin. Note the failure of Cap-G^NM^-EGFP to localize to prophase chromatin, while it is readily detected on metaphase and anaphase chromatin. Scale bar is 20 µm.
(TIF)

Figure S9 Functionality of *Cap-H2* transgenes. *UASP1-EGFP-Cap-H2* and *UASP1-mCherry-Cap-H2* were expressed in stage 10 egg chambers using the *mat αtub-GAL4* driver (C, D, c, d) or in 3^rd^ instar salivary glands using the *F4-GAL4* driver line (G, g, H, h). *Cap-H2* mutants retain the polytene chromosome morphology of nurse cell nuclei in stage 10 egg chambers which is normally lost in mid-oogenesis [24](Compare B, b with A, a). Expression of the *Cap-H2* transgenes at least partially restores the dispersal of the chromosomes when expressed in the *Cap-H2* mutant background (C, c, D, d). Conversely, *Cap-H2* overexpression results in the dispersal of the polytene salivary gland nuclei chromosomes (compare F, f with E, e). Likewise, ectopic expression of *EGFP-Cap-H2* (G,g) or *mCherry-Cap-H2* (H, h) results in the dispersal of the polytene salivary gland nuclei chromosomes. The *Cap-H2* alleles used were *Cap-H2^Df(3R)Exel6159^* (Exel6159), *Cap-H2^EY09979^* (EY09979), and *Cap-H2^TH2^* (TH2). (a–h) show representative, enlarged single nuclei from (A–H), respectively. Scale bars in A–D, E–H, and a–h are 20 µm, 50 µm, and 5 µm, respectively.
(TIF)

Figure S10 Absence of condensin II-like phenotypes in egg chambers of Cap-G^NM^ rescued females. Ovaries were prepared from *Cap-D3^EY00456^* homozygous females (A) and from *Cap-G* trans-heterozygous mutant females expressing *Cap-G^NM^* (*Cap-G^1^/Cap-G^6^; Cap-G^NM^*) or from sibling females (*Cap-G^1^/CyO; Cap-G^NM^*) (B). DNA was stained with Hoechst 33258 and the polytenic state of the nurse cell chromosomes was analyzed. Overviews of typical stage 6 and stage 10 egg chambers are shown in the left panels, while representative nurse cell nuclei for stage 6 and stage 10 egg chambers are shown in the right panels. Note the more condensed, polytene-like appearance of the nurse cell chromosomes in *Cap-D3* mutant egg chambers, while chromatin is more dispersed in egg chambers of Cap-G^NM^ rescued and control females. (C) Stage 10 egg chambers from *Cap-G* mutant females rescued by *da-Gal4* driven expression of *UASP1-Cap-G^NM^-EGFP* (upper panel) or from individuals expressing *Cap-G^FL^-EGFP* in a wild-type background (lower panel). Ovarioles were fixed, treated with Hoechst 33258 to stain DNA, and the localization of the transgene products was assessed by observing EGFP autofluorescence. Note that Cap-G^NM^-EGFP is excluded from the nuclei and is enriched in the cytoplasm, while Cap-G^FL^-EGFP co-localizes with nurse cell and follicle cell chromatin. In the merged panels, DNA is shown in red and EGFP autofluorescence in green. Scale bar units are in µm.
(TIF)

Table S1 Results of rescue experiments using various *Cap-G^FL^* and *Cap-G^NM^* transgene insertions. All rescued individuals were trans-heterozygous for the alleles *Cap-G^1^* and *Cap-G^6^*. UAS transgenes were expressed using the ubiquitous driver *da-GAL4*. The genomic transgenes were all pattB-constructs inserted at 96E. Crosses were kept at 22°C to 24°C. n.d. - not determined. 1) only very vew progeny were obtained from this cross. It was not possible to establish a rescue stock. 2) Rescued females gave rise to only very few progeny. 3) Only females rescued by one transgene copy were obtained in these crosses.
(DOCX)

Table S2 Complete list of proteins identified in anti-mRFP1 immunoprecipitates from ovary extracts of *Cap-G-mRFP1* expressing females. Condensin subunits are highlighted in light grey. The hits are ranked according to their cumulated peptide intensities in the Cap-G-mRFP1 sample. Included are the peptide intensities of the same proteins detected in precipitates from *w^1^*-ovaries not expressing *Cap-G-mRFP1*.
(XLSX)

Table S3 Complete list of proteins identified in anti-mRFP1 immunoprecipitates from embryo extracts of *Cap-G-mRFP1* individuals. Condensin subunits are highlighted in light grey. The hits are ranked according to their cumulated peptide intensities.
(XLSX)

Table S4 Complete list of proteins identified in anti-mRFP1 and anti-EGFP immunoprecipitates from ovary extracts of females expressing *mCherry-Cap-H2* or *EGFP-Cap-H2*, respectively. Condensin subunits are highlighted in light grey. The hits are ranked according to their cumulated peptide intensities.
(XLSX)

Table S5 Complete list of proteins identified in anti-EGFP immunoprecipitates from embryo extracts of *SMC2_h_-EGFP* expressing individuals. Condensin subunits are highlighted in light grey. The hits are ranked according to their cumulated peptide intensities.
(XLSX)

Table S6 Complete list of proteins identified in anti-SMC2 immunoprecipitates from *w^1^* embryo extracts. Condensin subunits are highlighted in light grey. The hits are ranked according to their cumulated peptide intensities.
(XLSX)

Table S7 Complete list of proteins identified in anti-EGFP immunoprecipitates from ovary extracts of *SMC2_h_-EGFP* expressing females. Condensin subunits are highlighted in light grey. The hits are ranked according to their cumulated peptide intensities.
(XLSX)

Table S8 Complete list of proteins identified in mock immunoprecipitates from *w^1^*-embryos. The hits are ranked according to their cumulated peptide intensities.
(XLSX)

Video S1 Embryo co-expressing EGFP-Cap-D2 and His2Av-mRFP1 progressing through mitosis 12. The left panel shows EGFP fluorescence only and the right panel the merge of the EGFP and mRFP1 channels. Time stamp is in min∶sec.
(MOV)

Video S2 Embryo co-expressing SMC2_h_-EGFP and His2Av-mRFP1 progressing through mitosis 12. The left panel shows EGFP fluorescence only and the right panel the merge of the EGFP and mRFP1 channels. Time stamp is in min∶sec.
(MOV)

Video S3 Embryo co-expressing Cap-G^FL^-EGFP and His2Av-mRFP1 progressing through mitosis 12. The left panel shows EGFP fluorescence only and the right panel the merge of the EGFP and mRFP1 channels. Time stamp is in min∶sec.
(MOV)

Video S4 Embryo expressing Cap-G^C^-EGFP progressing through mitosis 14. Time stamp is in min∶sec.
(MOV)

Video S5 Embryo co-expressing Cap-G^NM^-EGFP and His2Av-mRFP1 progressing through mitosis 14. The left panel shows EGFP fluorescence only and the right panel the merge of the EGFP and mRFP1 channels. Time stamp is in min∶sec.
(MOV)
