# Supplementary figures and images for: Functional Dissection of the Drosophila melanogaster Condensin Subunit Cap-G Reveals Its Exclusive Association with Condensin I
Source: PLoS Genet. 2013 Apr 18;9(4):e1003463. doi: 10.1371/journal.pgen.1003463 (PMC3630105; doi:10.1371/journal.pgen.1003463)

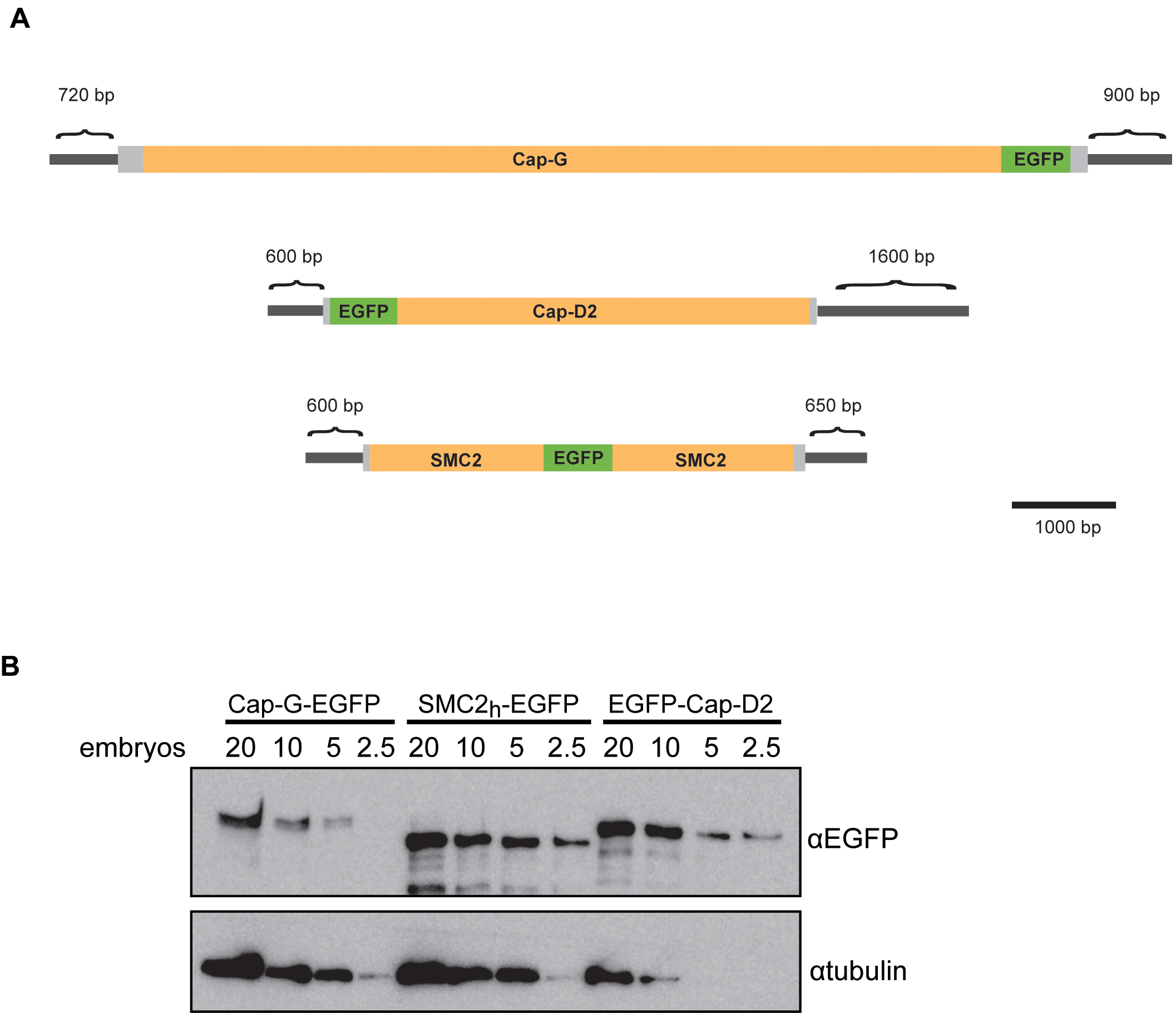

Supplement: Figure S1 — Construction and expression of EGFP-fused condensin variants. (A) Schematic presentation of all constructs, which are based on genomic DNA sequences. Orange bars represent the condensin reading frames including introns and the green bars the fused EGFP reading frame. Light grey bars indicate 5′- and 3′-UTRs. Dark grey bars represent 5′- and 3′-flanking genomic regions. While Cap-G and Cap-D2 were tagged at their C-terminus and N-terminus, respectively, the EGFP reading frame was inserted into SMC2 between the codons for amino acids glycine 582 and serine 583. (B) Analysis of expression levels. Extracts were prepared from 0–3 hrs old embryos derived from mothers carrying one transgene copy and which had been mated with wild type (w1) males. Proteins contained in four serial dilutions of each extract were separated by SDS-PAGE, blotted, and detected with monoclonal anti-EGFP antibodies (αEGFP) and anti-α-tubulin antibodies (αtubulin) as loading control. (TIF) [file pgen.1003463.s001.tif]

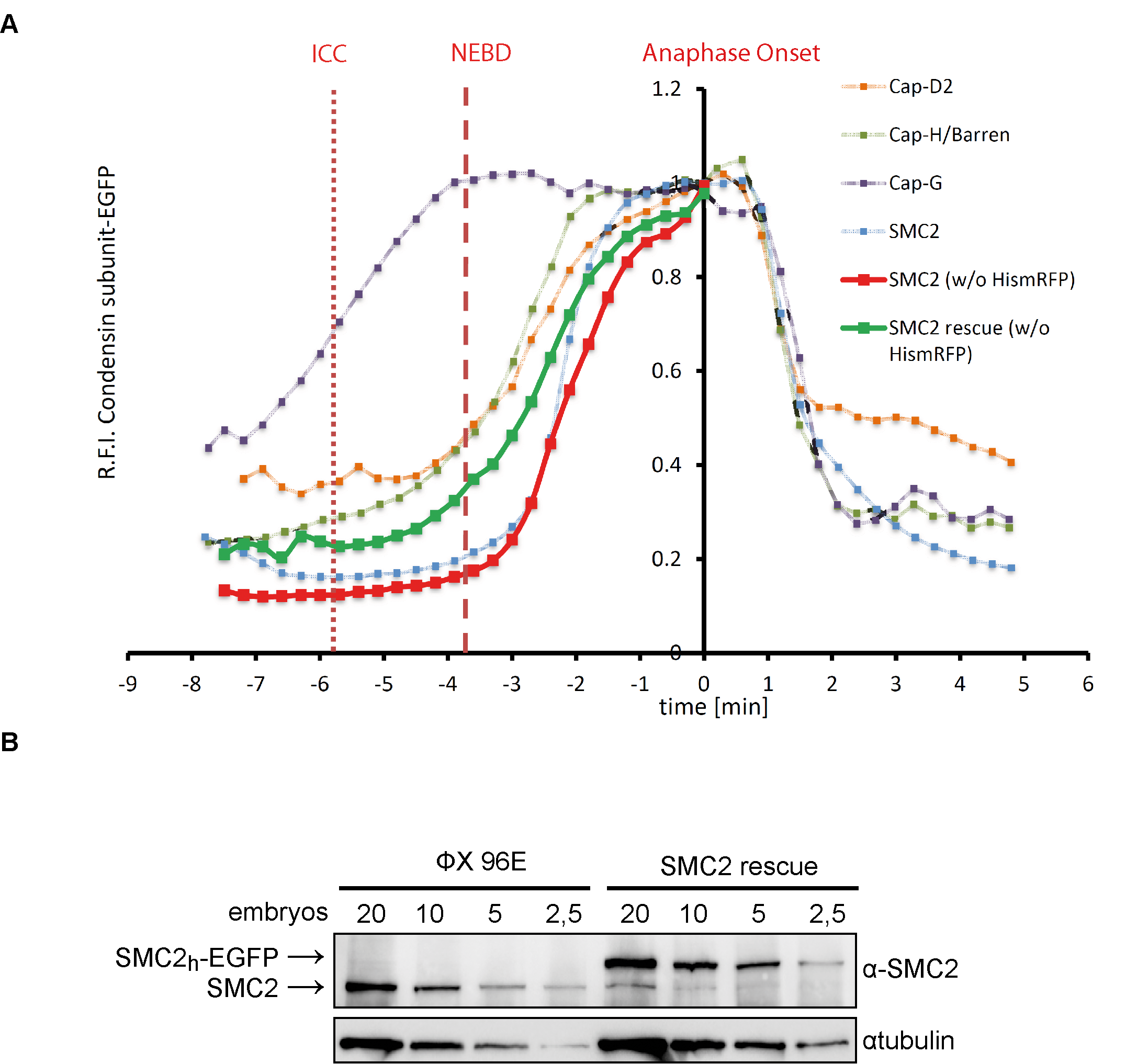

Supplement: Figure S2 — Dynamics of chromatin association of SMC2h-EGFP in an SMC2 mutant background. (A) The fluorescence intensity of SMC2h-EGFP in syncytial embryos laid by mothers with the genotype SMC2f06842/SMC2Df(2R)BSC429; gSMC2h-EGFPΦX-96E was determined for selected nuclei progressing through mitosis 12 in each frame, and is plotted as relative intensity per nucleus (green curve, SMC2 rescue). As these embryos did not contain the red fluorescent His2Av-mRFP1, a correction for chromatin compaction (as done in Figure 1B) was not possible. Thus, the data for SMC2h-EGFP in a SMC2+-background was processed analogously (red curve, SMC2 (w/o HismRFP)). Data series were aligned accordingly to anaphase onset (t0 = last metaphase frame). Data sets from a total of 28 nuclei from 12 embryos were aligned. The curves for Cap-D2, Cap-H/Barren, Cap-G, and SMC2 are the same as in Figure 1B and are shown for reference. The times of initiation of chromatin condensation (ICC) and NEBD are indicated by the dotted and dashed red lines, respectively. (B) Western blot analysis of extracts from 0–3 hrs old embryos with the genotype y1, M{vas-int.Dm}ZH-2A, w*; M{3xP3-RFP.attP′}ZH-96E (ΦX 96E) or laid by mothers with the genotype SMC2f06842/SMC2Df(2R)BSC429; gSMC2h-EGFPΦX-96E (SMC2 rescue). The blot was probed with anti-SMC2 antibodies (upper panel) and anti-α-tubulin antibodies as loading control (bottom panel). (TIF) [file pgen.1003463.s002.tif]

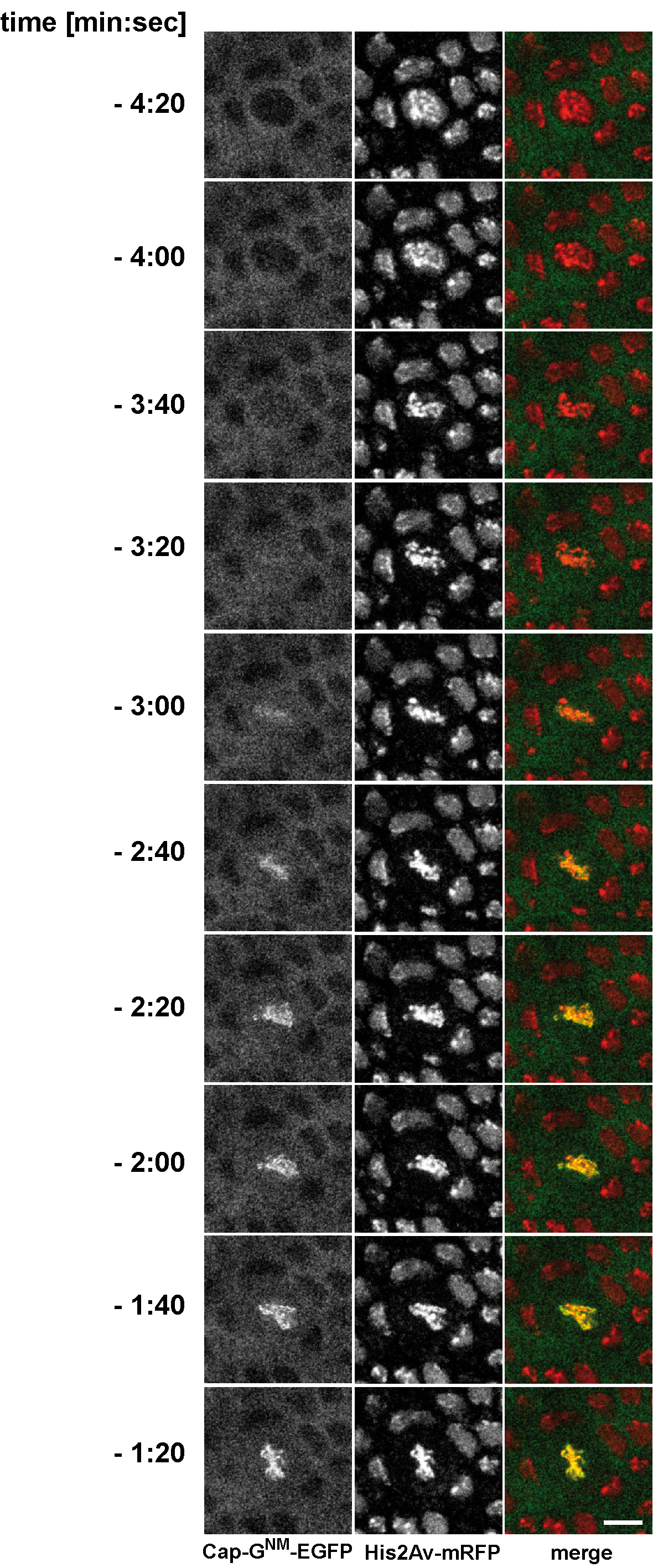

Supplement: Figure S3 — Time-lapse analysis of Cap-GNM-EGFP association with chromatin. Subcellular localization and chromatin association of Cap-GNM-EGFP observed in a living embryo progressing through epidermal mitosis 14. Expression of the UAST-Cap-GNM-EGFP transgene was driven by α4-tub-GAL4-VP16. NEBD occurs between time points −4∶00 and −3∶40 as indicated by the influx of Cap-GNM-EGFP into the nuclear space. Chromatin enrichment of Cap-GNM-EGFP is detectable starting from time point -3∶20. Individual frames of time lapse movies are shown with time points indicated in min∶sec (t = 0, anaphase onset). In the merged panels, His2Av-mRFP and Cap-GNM-EGFP are shown in red and green, respectively. Scale bar is 5 µm. (TIF) [file pgen.1003463.s003.tif]

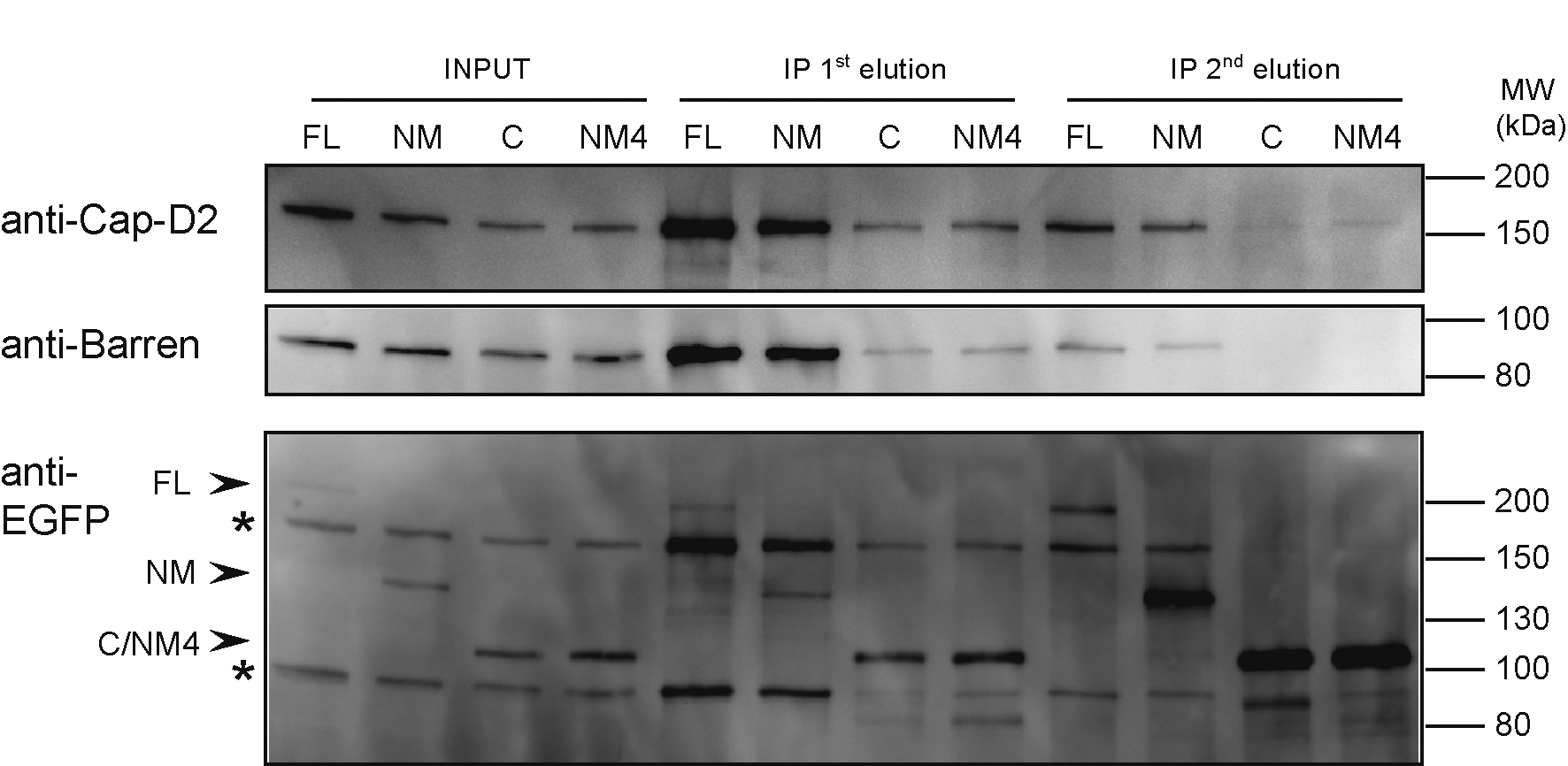

Supplement: Figure S4 — Western blot analysis of proteins associated with Cap-G fragments. Extracts from 3–6 h old embryos expressing various EGFP-fused Cap-G-fragments driven by α4-tub-GAL4-VP16 were subjected to immunoprecipitation with rabbit-anti-EGFP antibodies. Bound proteins were eluted in two steps with increasing stringency. Precipitates were separated by SDS-PAGE and blotted onto a nitrocellulose membrane. The blot was probed with antibodies against Cap-D2 (top panel), against Cap-H/Barren (middle panel) and against EGFP (lower panel). Cap-D2 and Cap-H/Barren were efficiently precipitated by Cap-GFL-EGFP (FL) and Cap-GNM-EGFP (NM) and were eluted during the first step (IP 1st elution), while they were much less efficiently precipitated by Cap-GNM4-EGFP (NM4) and Cap-GC-EGFP (C). The second elution step (IP 2nd elution) mainly reveals the recovery of the EGFP-fused Cap-G-fragments (indicated by arrowheads). Note that Cap-GNM4-EGFP and Cap-GC-EGFP migrate at the same position in the SDS-polyacrylamide gel. Asterisks indicate proteins cross-reacting with the anti-EGFP antibody. (TIF) [file pgen.1003463.s004.tif]

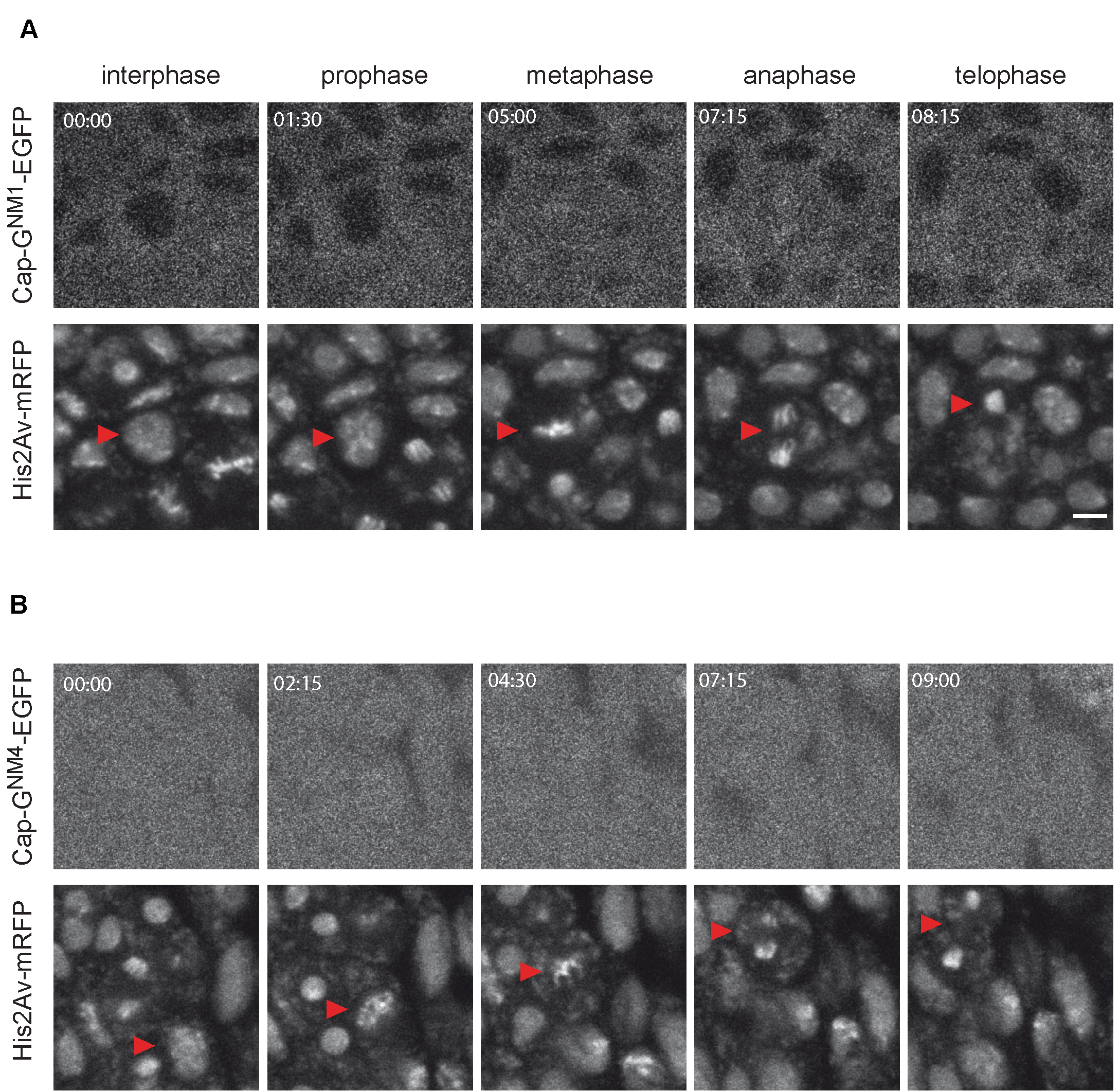

Supplement: Figure S5 — Subcellular localization of Cap-GNM1-EGFP and Cap-GNM4-EGFP. Living embryos expressing His2Av-mRFP1 and Cap-GNM1-EGFP (A) or Cap-GNM4-EGFP (B) were observed while progressing through epidermal mitosis 14. Individual frames of time lapse movies are shown with time points indicated in min∶sec. The top rows show the distribution of the EGFP-fused Cap-G fragments and the bottom rows of His2Av-mRFP1. Red triangles highlight individual cells progressing through mitosis. Scale bar is 5 µm. (TIF) [file pgen.1003463.s005.tif]

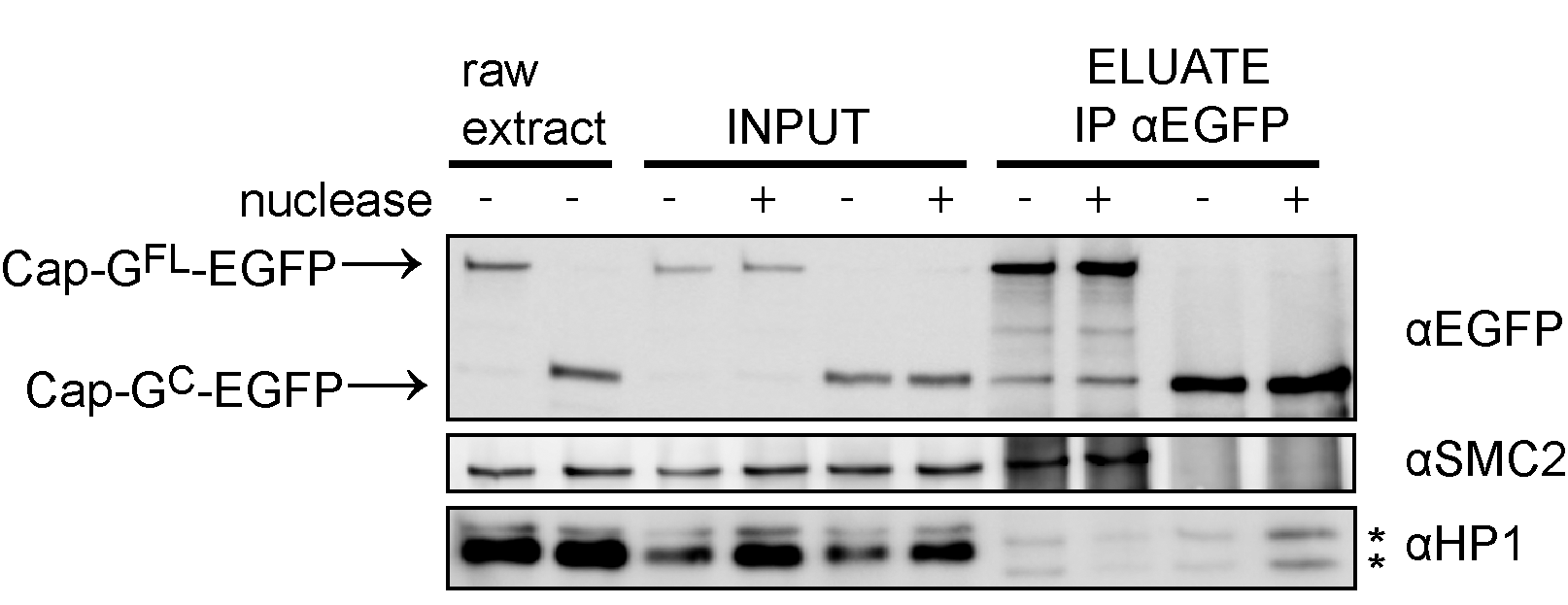

Supplement: Figure S6 — HP1 does not co-precipitate with Cap-G. Extracts of 4–7 hrs old embryos expressing UAS-Cap-GFL-EGFP or UAS-Cap-GC-EGFP driven by da-GAL4 were either treated (+) or not treated (−) with nuclease to solubilize chromatin. After centrifugation to pellet debris and undigested chromatin, the supernatant was used for immunoprecipitation with monoclonal anti-EGFP antibodies. Proteins in the raw extracts, the cleared supernatants (INPUT) and the eluates after immunoprecipitation (ELUATE IP αEGFP) were separated by SDS-PAGE, blotted, and detected using a different anti-EGFP antibody, anti-SMC2, and anti-HP1 antibodies. Asterisks denote cross-reactions of the HP1-antibody. HP1 is not detectable in the IP eluates, while SMC2 is readily and specifically co-precipitated with Cap-GFL. Note that in the INPUT samples treated with nuclease, the abundance of HP1 is increased when compared with the samples without nuclease treatment, indicating successful chromatin solubilization. (TIF) [file pgen.1003463.s006.tif]

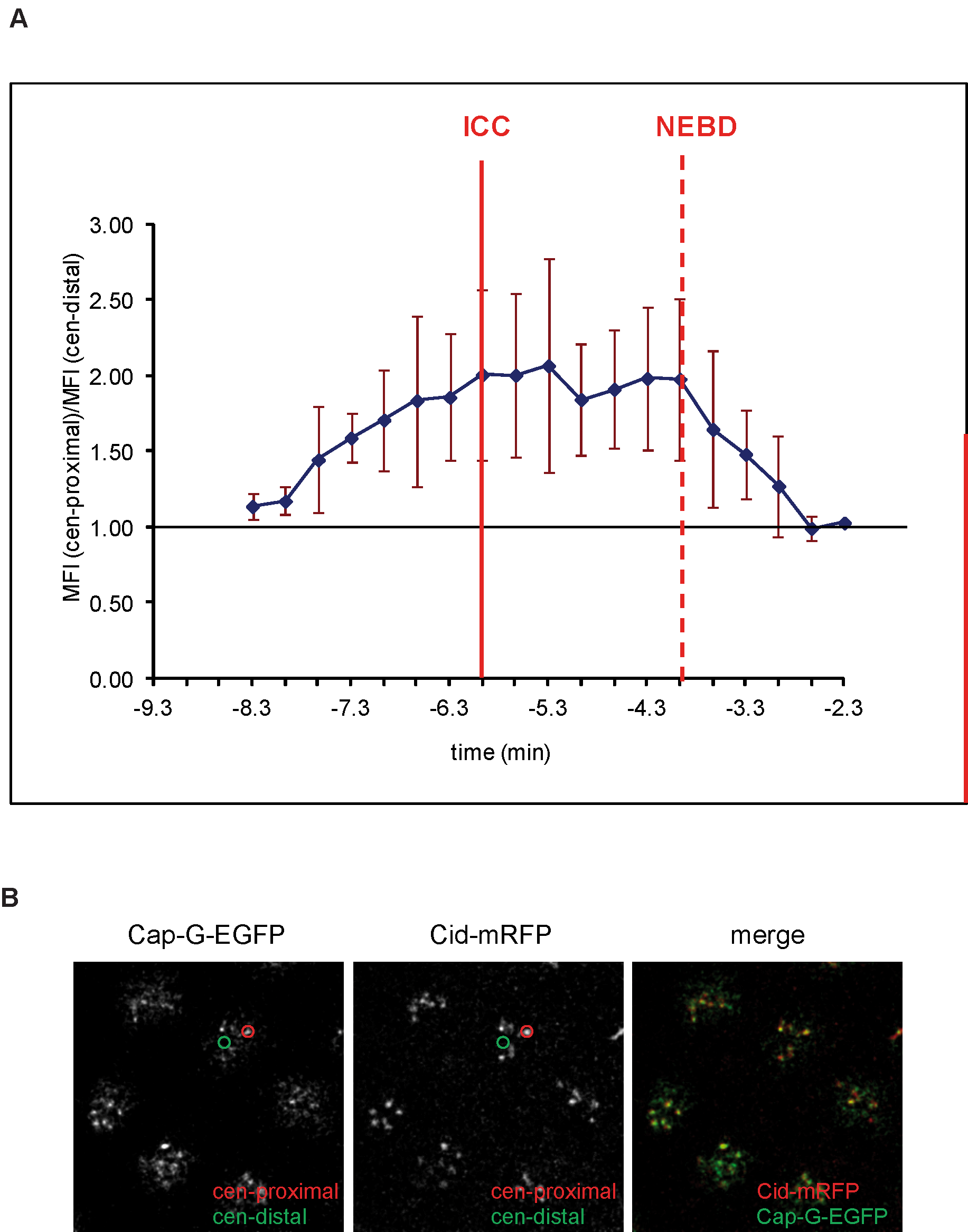

Supplement: Figure S7 — Quantitative measurement of Cap-G-EGFP accumulation at centromeric regions. Embryos co-expressing Cap-GFL-EGFP and Cid-mRFP1 were observed while progressing through syncytial mitosis 12. (A) Graphic representation of the ratios between mean Cap-G-EGFP fluorescence intensity of centromere proximal regions (MFI cen-proximal) and the mean Cap-G-EGFP fluorescence intensity of centromere distal regions (MFI cen-distal) plotted over time. t = 0 min corresponds to anaphase onset. n = 62 for each time point. ICC (initiation of chromosome condensation) and NEBD (nuclear envelope breakdown) time points are adapted from the experiment shown in Figure 1. (B) Example of images illustrating the selection of R.O.I.s for the quantitative fluorescence measurements. (TIF) [file pgen.1003463.s007.tif]

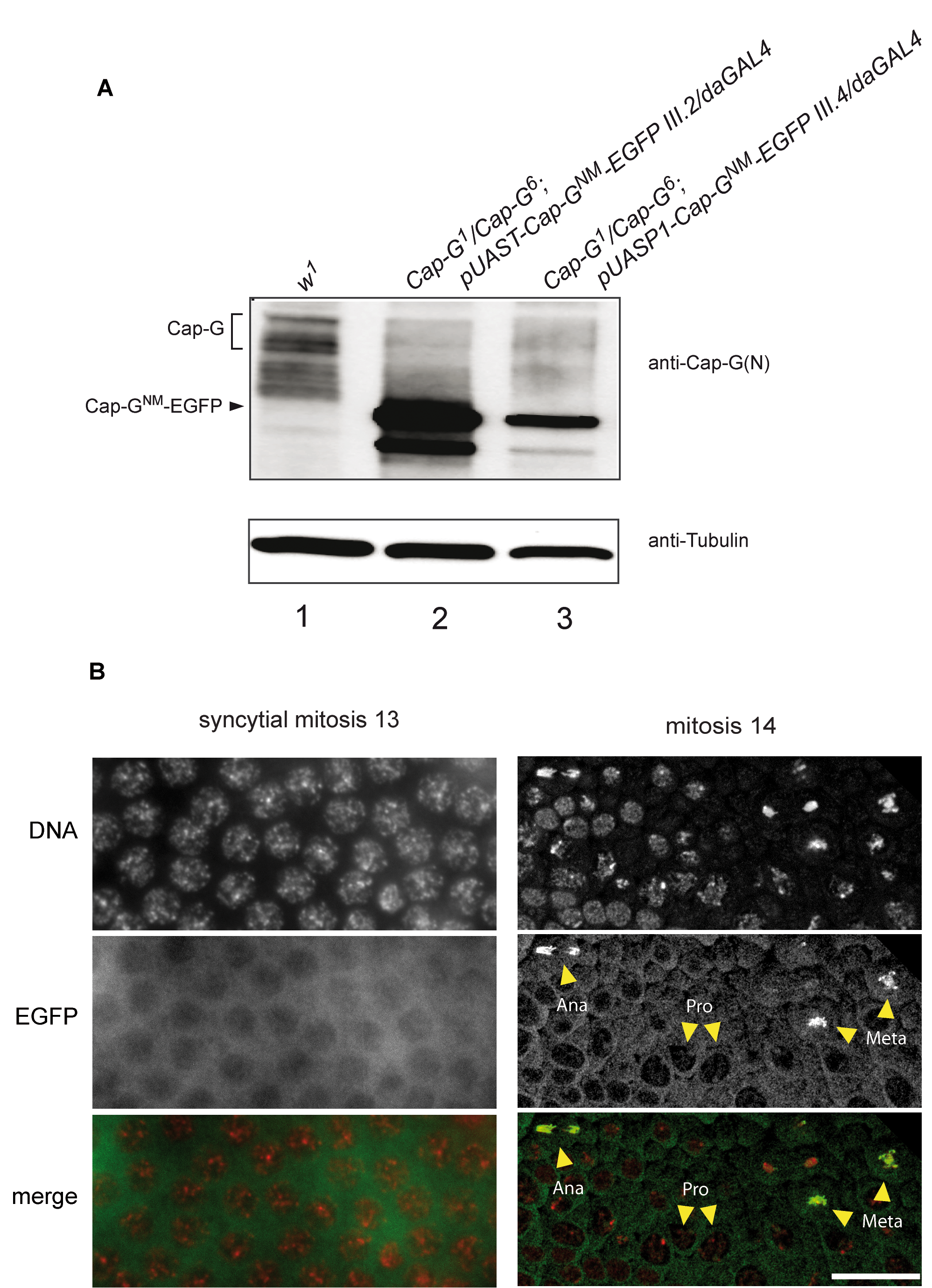

Supplement: Figure S8 — Cap-G mutant animals rescued by Cap-GNM-EGFP: Lack of endogenous Cap-G expression and failure of Cap-GNM-EGFP to localize to prophase chromatin. (A) Extracts were prepared from ovaries from animals expressing wild type Cap-G (w1, lane 1) or the C-terminally truncated variant Cap-GNM-EGFP in a Cap-G1/Cap-G6 trans-heterozygous mutant background (lanes 2 and 3) under control of the ubiquitous driver da-GAL4. The transgenes were contained either in the vector pUAST (lane 2) or in the vector pUASP1 (lane 3). Extracts corresponding to 5 ovaries were separated on an SDS-polyacrylamide gel, blotted onto a nitrocellulose membrane and the blot was probed with anti-Cap-G antibodies recognizing the N-terminus of the protein (anti-Cap-G(N)) or as a loading control with anti-tubulin antibodies. Note that full-length Cap-G is partially degraded in lane 1. (B) Embryos derived from mothers with the genotype Cap-G1/Cap-G6; da-GAL4/UASP1-Cap-GNM-EGFP III.4 were fixed and treated with Hoechst 33258 to stain the DNA. In the left panel, nuclei in the periphery of an embryo are shown progressing through prophase of syncytial mitosis 13. The right panel shows epidermal nuclei of an embryo progressing through mitosis 14. Cells in prophase (Pro), metaphase (Meta) and anaphase (Ana) are indicated by yellow arrowheads. Prophase cells were identified by the appearance of condensed chromatin. Note the failure of Cap-GNM-EGFP to localize to prophase chromatin, while it is readily detected on metaphase and anaphase chromatin. Scale bar is 20 µm. (TIF) [file pgen.1003463.s008.tif]

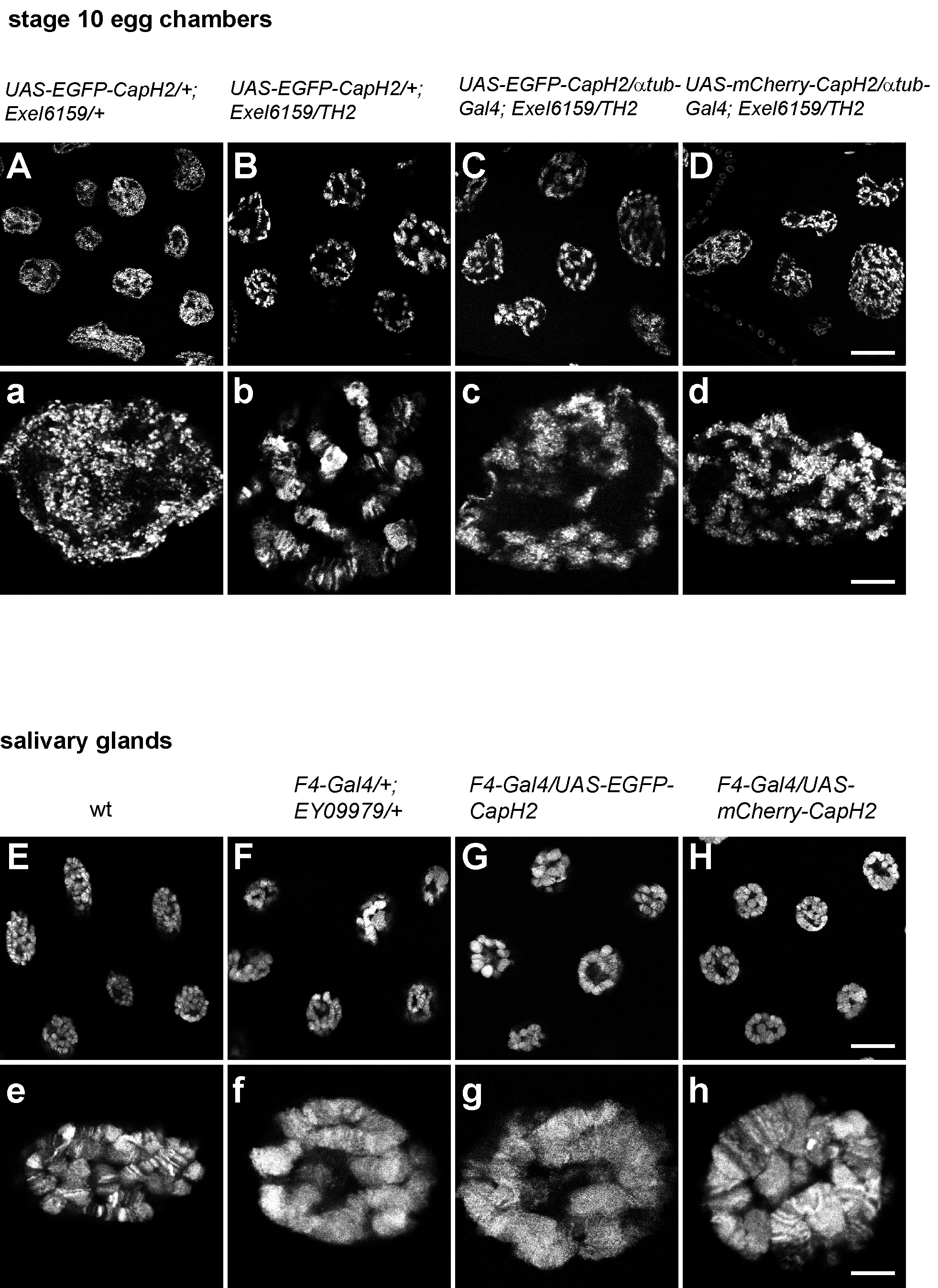

Supplement: Figure S9 — Functionality of Cap-H2 transgenes. UASP1-EGFP-Cap-H2 and UASP1-mCherry-Cap-H2 were expressed in stage 10 egg chambers using the mat αtub-GAL4 driver (C, D, c, d) or in 3rd instar salivary glands using the F4-GAL4 driver line (G, g, H, h). Cap-H2 mutants retain the polytene chromosome morphology of nurse cell nuclei in stage 10 egg chambers which is normally lost in mid-oogenesis [24](Compare B, b with A, a). Expression of the Cap-H2 transgenes at least partially restores the dispersal of the chromosomes when expressed in the Cap-H2 mutant background (C, c, D, d). Conversely, Cap-H2 overexpression results in the dispersal of the polytene salivary gland nuclei chromosomes (compare F, f with E, e). Likewise, ectopic expression of EGFP-Cap-H2 (G,g) or mCherry-Cap-H2 (H, h) results in the dispersal of the polytene salivary gland nuclei chromosomes. The Cap-H2 alleles used were Cap-H2Df(3R)Exel6159 (Exel6159), Cap-H2EY09979 (EY09979), and Cap-H2TH2 (TH2). (a–h) show representative, enlarged single nuclei from (A–H), respectively. Scale bars in A–D, E–H, and a–h are 20 µm, 50 µm, and 5 µm, respectively. (TIF) [file pgen.1003463.s009.tif]

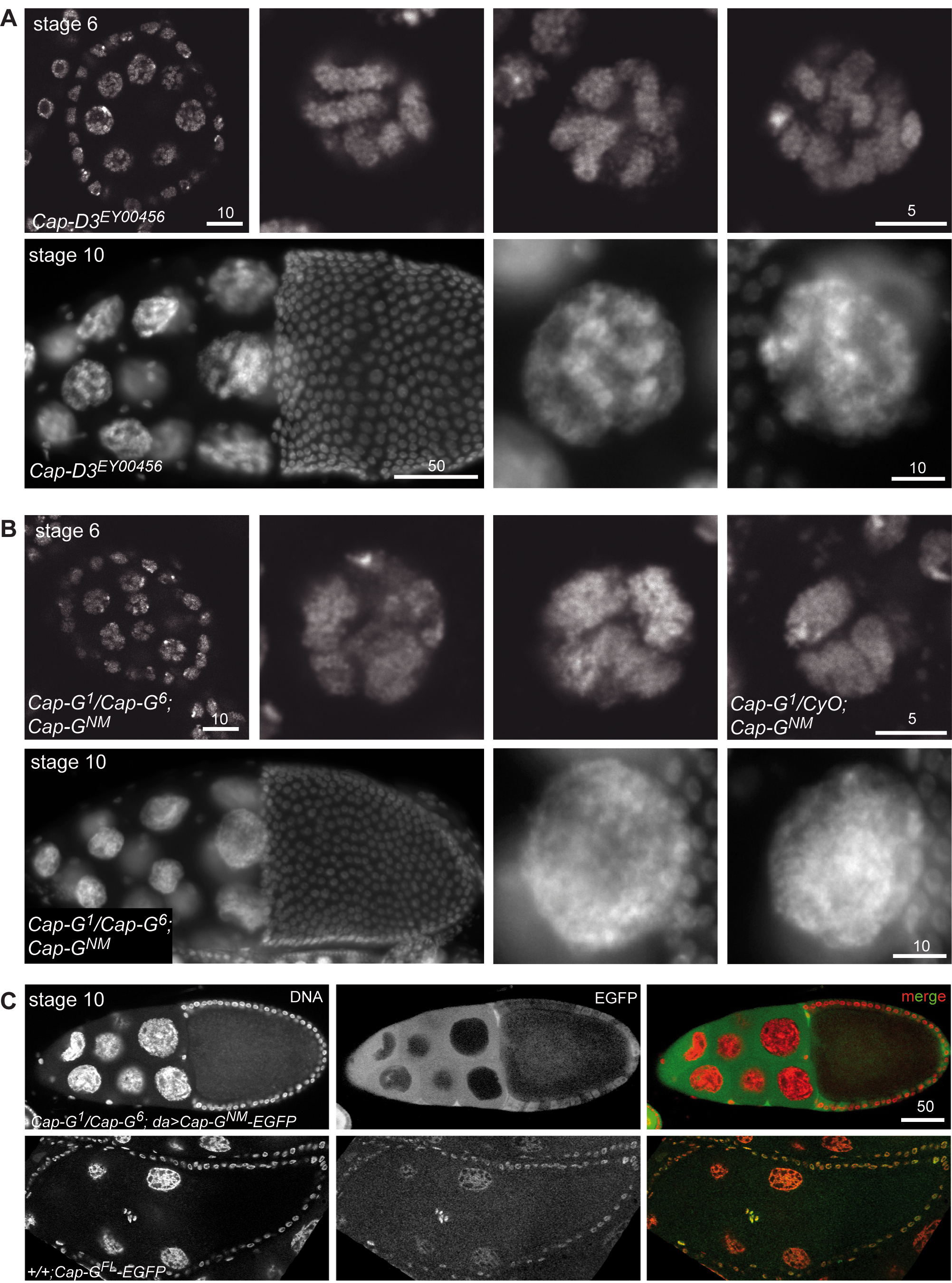

Supplement: Figure S10 — Absence of condensin II-like phenotypes in egg chambers of Cap-GNM rescued females. Ovaries were prepared from Cap-D3EY00456 homozygous females (A) and from Cap-G trans-heterozygous mutant females expressing Cap-GNM (Cap-G1/Cap-G6; Cap-GNM) or from sibling females (Cap-G1/CyO; Cap-GNM) (B). DNA was stained with Hoechst 33258 and the polytenic state of the nurse cell chromosomes was analyzed. Overviews of typical stage 6 and stage 10 egg chambers are shown in the left panels, while representative nurse cell nuclei for stage 6 and stage 10 egg chambers are shown in the right panels. Note the more condensed, polytene-like appearance of the nurse cell chromosomes in Cap-D3 mutant egg chambers, while chromatin is more dispersed in egg chambers of Cap-GNM rescued and control females. (C) Stage 10 egg chambers from Cap-G mutant females rescued by da-Gal4 driven expression of UASP1-Cap-GNM-EGFP (upper panel) or from individuals expressing Cap-GFL-EGFP in a wild-type background (lower panel). Ovarioles were fixed, treated with Hoechst 33258 to stain DNA, and the localization of the transgene products was assessed by observing EGFP autofluorescence. Note that Cap-GNM-EGFP is excluded from the nuclei and is enriched in the cytoplasm, while Cap-GFL-EGFP co-localizes with nurse cell and follicle cell chromatin. In the merged panels, DNA is shown in red and EGFP autofluorescence in green. Scale bar units are in µm. (TIF) [file pgen.1003463.s010.tif]
