# Supplementary material for: Functional Dissection of the Drosophila melanogaster Condensin Subunit Cap-G Reveals Its Exclusive Association with Condensin I
Source: PLoS Genet. 2013 Apr 18;9(4):e1003463. doi: 10.1371/journal.pgen.1003463 (PMC3630105; doi:10.1371/journal.pgen.1003463)
Supplement: Table S1 — Results of rescue experiments using various Cap-GFL and Cap-GNM transgene insertions. All rescued individuals were trans-heterozygous for the alleles Cap-G1 and Cap-G6. UAS transgenes were expressed using the ubiquitous driver da-GAL4. The genomic transgenes were all pattB-constructs inserted at 96E. Crosses were kept at 22°C to 24°C. n.d. - not determined. 1) only very vew progeny were obtained from this cross. It was not possible to establish a rescue stock. 2) Rescued females gave rise to only very few progeny. 3) Only females rescued by one transgene copy were obtained in these crosses. (DOCX) [file pgen.1003463.s011.docx]

| Transgene | Number of transgene copies | n | Rescue (% of expected) | Fertility (*inter se*) | Fertility (x *w^1^*) |
| --- | --- | --- | --- | --- | --- |
| UAST-Cap-G^FL^-EGFP III.3 | 1 | 345 | 7.4 | - | + (only males) |
| UAST-Cap-G^NM^-EGFP III.1 | 1 | 750 | 80.0 | - | + (only males) |
| UAST-Cap-G^NM^-EGFP III.2 | 1 | 460 | 82.6 | - | + (only males) |
| UASP1-Cap-G^NM^-EGFP III.4 | 1 | 1255 | 61.9 | - | + (both sexes) |
| genomic Cap-G^FL^-EGFP III.1 | 1 | 1575 | 45.9 | - | - |
|  | 2 | n.d. | n.d. | +^1^ | + (both sexes)^2^ |
| genomic Cap-G^NM^-EGFP III.1 | 1 | 1010 | 18.4^3^ | - | - |
|  | 2 |  | 27.9 | - | - |
| genomic Cap-G^NM^ III.2 | 1 | 998 | 26.1^3^ | - | - |
|  | 2 |  | 43.2 | - | + (only males) |
